# Boundedness, persistence and stability for classes of forced difference equations arising in population ecology

**DOI:** 10.1007/s00285-019-01388-7

**Published:** 2019-06-06

**Authors:** D. Franco, C. Guiver, H. Logemann, J. Perán

**Affiliations:** 1 Departamento de Matemática Aplicada, E.T.S.I. Industriales, Universidad Nacional de Educación a Distancia (UNED), c/ Juan del Rosal 12, 28040 Madrid, Spain; 2 Department of Mathematical Sciences, University of Bath, Claverton Down, Bath, BA2 7AY UK

**Keywords:** Absolute stability, Density-dependent population models, Environmental forcing, Forced systems, Global asymptotic stability, Infinite-dimensional systems, Input-to-state stability, Integral projection models, Lur’e systems, Population persistence, 37N25, 37N35, 39A22, 39A30, 47B65, 47N60, 93D09, 93D15

## Abstract

Boundedness, persistence and stability properties are considered for a class of nonlinear, possibly infinite-dimensional, forced difference equations which arise in a number of ecological and biological contexts. The inclusion of forcing incorporates the effects of control actions (such as harvesting or breeding programmes), disturbances induced by seasonal or environmental variation, or migration. We provide sufficient conditions under which the states of these models are bounded and persistent uniformly with respect to the forcing terms. Under mild assumptions, the models under consideration naturally admit two equilibria when unforced: the origin and a unique non-zero equilibrium. We present sufficient conditions for the non-zero equilibrium to be stable in a sense which is strongly inspired by the input-to-state stability concept well-known in mathematical control theory. In particular, our stability concept incorporates the impact of potentially persistent forcing. Since the underlying state-space may be infinite dimensional, our framework enables treatment of so-called integral projection models (IPMs). The theory is applied to a number of examples from population dynamics.

## Introduction

We consider boundedness, persistence and stability properties of the following class of forced difference equations

1.1 $$\begin{aligned} x^\nabla =Ax+bf(u,c^*x)+v ,\quad x(0)=x^0\,, \end{aligned}$$

where $x^\nabla$ denotes the image of *x* under the left shift operator, that is, $x^\nabla (t) = x(t+1)$ for all nonnegative integers *t*. The difference equation (1.1) comprises a linear component *Ax*, where *A* is a bounded linear operator on the state-space *X*, assumed to be a Banach space, and a nonlinear component $b f(u,c^* x)$. Here $b \in X$ and $c^* \in X^*$, the dual space of *X*, and *f* is a (typically nonlinear) real-valued function that may depend on a variable *u* which, along with *v*, denotes forcing. Depending on the context, *u* and *v* are interpreted as a control, input or disturbance. Full details are given in Sect. 3.

Our motivation for studying (1.1), and particularly the properties of boundedness, persistence and stability, is the potential for applications to population biology and theoretical ecology where models of the form (1.1) often arise. There is clear biological relevance for these three properties depending on the context. Boundedness is a necessary property of a sensible biological model and, moreover, is a key concept when seeking to understand the potential effects of an invasive species or mutant in a novel environment, see, for example, Eager et al. (2014). The notion of persistence relates to the survival of a population (or survival of certain stage-classes) and is relevant, for example, in the context of providing lower bounds for predicted yield in agriculture and horticulture. We comment that several persistence concepts and their properties have been studied in the literature (Franco et al. 2017; Schreiber 2012; Smith and Thieme 2011, 2013; Wen et al. 2016). Informally, boundedness and persistence are opposite properties, concerned with populations becoming neither too big, or too small, respectively. Stability is, of course, a fundamental consideration in all fields where dynamic modelling occurs and pertains to both the qualitative and quantitative long-term behaviour of solutions.

Since we seek to model a variety of structured populations, see for instance Caswell (2001), Cushing (1998), the dimension of the state-space *X* is not constrained. In fact, the case $\dim \,X = \infty$ is included in our development, and therefore, (1.1) can be used to model certain partial-difference and integro-difference equations. The latter, in form of so-called integral projection models, are often employed to model populations with a continuous structure or stage-parameter, in which case the state space *X* of (1.1) is naturally a space of functions. Furthermore, in a populations dynamics context, the state variable *x* must be nonnegative-valued to be biologically meaningful, corresponding to abundance, concentration or density, for example. Consequently, we impose suitable positivity assumptions on the model data *A*, *b*, $c^*$ and *f* implying that (1.1) is a positive dynamical system, that is, the dynamics leave a positive cone in *X* invariant. In this sense, the current paper is embedded in the research field of positive dynamical systems on which there exists a rich literature, see Berman et al. (1989), Haddad et al. (2010), Krasnosel’skij et al. (1989), Krause (2015), and Luenberger (1979), to mention a few references.

The dynamics of the unforced version of (1.1) (that is, $f(u,y)=f(y)$ and $v=0$) have recently been studied by several researchers in a variety of biologically motivated contexts (Eager 2016; Eager et al. 2014; Franco et al. 2014; Rebarber et al. 2012; Smith and Thieme 2013; Townley et al. 2012). Under certain assumptions, (1.1) admits a so-called “trichotomy of stability” where precisely one of three situations occurs: zero is globally asymptotically stable; there is a unique non-zero equilibrium which attracts all non-zero solutions; or, all non-zero solutions diverge asymptotically.

In the literature, the study of boundedness, persistence and stability properties of (1.1) does not usually include forcing [denoted by the terms *u* and *v* in (1.1)], with Franco et al. (2017) being an exception. As is well-known, classical (Lyapunov) stability notions are not applicable when exogenous, and potentially persistent, forcing is present, and forcing can have adverse, extreme and unintuitive effects on nonlinear dynamics, see, for example, Teel and Hespanha (2004). Forcing is very natural and important in a biological or ecological context, however, as it allows for the modelling of human interventions, such as management strategies, hunting, poaching, predation or farming, as well as uncontrolled seasonal or demographic variation, or migration. Mathematical systems and control theory provides a toolbox for the study of control and forcing in dynamical systems (Hinrichsen and Pritchard 2005; Sontag 2013). Moreover, models of the form (1.1) arise in mathematical control theory in which context they are often called Lur’e systems after the Soviet scientist A. I. Lur’e who made early contributions to their stability properties in the 1940s. The study of the stability properties of Lur’e systems constitutes absolute stability theory which, loosely speaking, seeks to conclude stability of the (unforced) system (1.1) through the interplay of frequency-domain properties of the function $c^*(sI-A)^{-1}b$ and boundedness or sector properties of the nonlinearity *f*, yielding readily checkable sufficient conditions for global asymptotic and exponential stability of the unforced system (1.1), see Haddad and Chellaboina (2008), Hinrichsen and Pritchard (2005), Khalil (2014), Liberzon (2006), Vidyasagar (1993) and Yakubovich et al. (2004). Furthermore, absolute stability is at the heart of Rebarber et al. (2012), Townley et al. (2012), where it is used to derive the above-mentioned stability trichotomy. Recent work Bill et al. (2016), Gilmore et al. (2019), Sarkans and Logemann (2015) and Sarkans and Logemann (2016a) has combined absolute stability ideas with input-to-state stability (ISS) theory to obtain stability criteria which apply to forced Lur’e systems. The ISS concept seeks to generalise the familiar estimate: there exist $M \ge 1$ and $\gamma \in (0,1)$ such that

1.2 $$\begin{aligned} \Vert x(t) \Vert \le M \big (\gamma ^t \Vert x(0)\Vert + \max _{0 \le \tau \le t-1} \Vert d (\tau )\Vert \big ),\quad \forall \,t \in {\mathbb {Z}}_+, \end{aligned}$$

valid for the forced linear system $x^\nabla = Ax + d$ with exponentially stable *A*, to forced nonlinear control systems. Note that (1.2) holds uniformly in *x*(0) and *d* and that, on the right-hand side of (1.2), the impact of the initial state *x*(0) decays over time, and the contributions of *x*(0) and the forcing *d* are separated. ISS was introduced by Sontag (1989) in 1989 and has, in the last 30 years, been developed into a mature stability theory of forced nonlinear control systems; see, for instance Dashkovskiy et al. (2011), Karafyllis and Jiang (2011) and Sontag (2006).

Starting with an ISS result from Gilmore et al. (2019), here we derive sufficient conditions for the state *x* of (1.1) to exhibit certain boundedness, persistence and stability properties. Our main results are Theorems 4.4 and 5.2, which address boundedness and persistence, and stability, respectively. The persistence notion employed is related to that in Smith and Thieme (2011, 2013), see Sect. 4, and the stability concept we consider is closely related to the above-mentioned ISS property, see Sect. 5. We present conditions under which the unforced system (1.1) admits, in addition to the zero equilibrium, a unique non-zero equilibrium which is both persistent and ISS (in suitable senses). A consequence of the stability property is the guarantee of asymptotic convergence of the state *x* when subject to convergent forcing (see statement (b) of Theorem 5.2 and Corollary 5.6). We remark that the theory developed in this paper is a far reaching generalization of our earlier paper (Franco et al. 2017), and has partial overlap with the works (Rebarber et al. 2012; Smith and Thieme 2013; Townley et al. 2012). We discuss the generalization of Franco et al. (2017), and compare our results to those in Rebarber et al. (2012), Smith and Thieme (2013), Townley et al. (2012), across Remarks 4.6 and 5.5.

The paper is organised as follows. In Sect. 2 we collect a number of mathematical preliminaries, and in Sect. 3 we provide more details on the the class of models (1.1). Sections 4 and 5 contain the main results pertaining to boundedness and persistence, and stability, respectively. Section 6 contains detailed discussions of both finite and infinite-dimensional examples. In particular, we provide a detailed discussion of two infinite-dimensional systems both of which involve integral projection models (Childs et al. 2003; Easterling et al. 2000; Ellner and Rees 2006; Merow et al. 2014; Rebarber et al. 2012). Some summarising remarks are made in Sect. 7. Proofs can be found in Appendix A, and a number of other technicalities have been relegated to Appendices B and C.

## Preliminaries

As usual let ${\mathbb {N}}$, ${\mathbb {Z}}$, ${\mathbb {R}}$ and ${\mathbb {C}}$ denote the positive integers (natural numbers), integers, real numbers and complex numbers, respectively. Furthermore,

$$\begin{aligned} {\mathbb {Z}}_+:=\big \{m\in {\mathbb {Z}}: m\ge 0\big \}={\mathbb {N}}\cup \{0\}\quad \text{ and }\quad {\mathbb {R}}_+:=\big \{r\in {\mathbb {R}}:r\ge 0\big \}\,. \end{aligned}$$

Let *Y* and *Z* be normed vector spaces. The space of all bounded linear operators $Y\rightarrow Z$ is denoted by ${\mathcal {L}}(Y,Z)$. As usual, we set ${\mathcal {L}}(Y):={\mathcal {L}}(Y,Y)$ and $Y^*:={\mathcal {L}}(Y,{\mathbb {F}})$, where ${\mathbb {F}}={\mathbb {R}}$ if *Y* is real and ${\mathbb {F}}={\mathbb {C}}$ if *Y* is complex. For $T\in {\mathcal {L}}(Y,Z)$, the adjoint operator $T^*\in {\mathcal {L}}(Z^*,Y^*)$ is defined by $(T^*z^*)(y):=(z^*T)(y)$ for all $y\in Y$ and $z^*\in Z^*$.

The open ball centred at $y\in Y$ of radius $\rho >0$ is defined by

$$\begin{aligned} {\mathbb {B}}(y,\rho ):=\big \{x\in Y: \Vert x-y\Vert <\rho \big \}. \end{aligned}$$

In this paper, the letters *X* and *C* will always denote a real Banach space and a cone in *X*, respectively. Recall that $C\subset X$ is a *cone* if *C* is closed, convex, $\rho C\subset C$ for all $\rho \ge 0$ and $C\cap (-C)=\{0\}$, where

$$\begin{aligned} \rho C:=\{\rho x: x\in C\}\quad \text{ and }\quad -C:=\{-x: x\in C\}\,. \end{aligned}$$

Note that the singleton $\{0\}$ is a cone and that $0\in C$. If $C\not =\{0\}$, then the cone *C* is said to be non-trivial. Furthermore, note that $\{\rho x+\sigma y: x,y\in C,\,\rho ,\sigma \ge 0\}\subset C$, as follows from convexity and the property that $\rho C\subset C$ for all $\rho \ge 0$. We say that *C* is *reproducing* if $X=C-C$, where $C-C:=\{x-y:x,y\in C\}$. If the closure of $C-C$ is equal to *X*, then *C* is said to be *total*.

We define a partial order on *X* by setting, for $x,y\in X$,

$$\begin{aligned} x\ge y\quad \text{ if }\quad x-y\in C\,. \end{aligned}$$

We write $x>y$ if $x\ge y$ and $x\not =y$. Furthermore, if $\mathrm{int}\,C\not =\emptyset$ and $x-y\in \mathrm{int}\,C$, then $x>y$ (because otherwise $0\in \mathrm{int}\,C$, in which case there exists $\rho >0$ such that ${\mathbb {B}}(0,\rho )\subset C$ and thus ${\mathbb {B}}(0,\rho )\subset C\cap (-C)$ which is impossible).

Defining $$C^*\subset X^*$$ by

$$\begin{aligned} C^*:=\big \{x^*\in X^*:x^*(x)\ge 0\,\,\text{ for } \text{ all } x\in C \big \}\,, \end{aligned}$$

then it is straightforward to verify that $C^*$ satisfies all the properties of a cone except the condition $C^*\cap (-C^*)=\{0\}$. It is well known (Deimling 1985, p. 221) and not difficult to show that $C^*$ is a cone if, and only if, *C* is *total*, in which case $C^*$ is called *dual cone* of *C*.

For the rest of the paper it will always be assumed that the cone *C* is total, and so, $C^*$ is a cone in $X^*$.

Note that if $x,y\in C$, $x^*\in C^*$ and $x\ge y$, then $x^*(x)\ge x^*(y)$. The partial order on $X^*$ induced by $C^*$ will also be denoted by “ $\ge$ ”, that is, for $x^*,y^*\in X^*$, we write $x^*\ge y^*$ if $x^*-y^*\in C^*$. It is clear that, for $x^*,y^*\in X^*$, we have $x^*\ge y^*$ if, and only if, $x^*(x)\ge y^*(x)$ for all $x\in C$.

In the following we will be interested in scenarios wherein $\mathrm{int}\,C^*\not =\emptyset$. The first statement of the next lemma provides a characterization of elements in $\mathrm{int}\,C^*$.

### Lemma 2.1

Let $$x^*\in X^*$$.

(a): The functional $x^*$ is in $\mathrm{int}\,C^*$ if, and only if,
  2.1 $$\begin{aligned} \inf _{\xi \in C,\,\Vert \xi \Vert =1}x^*(\xi )>0\,. \end{aligned}$$
(b): If $x^*\in \mathrm{int}\,C^*$, $y^*\in C^*$ and $y^*\ge x^*$, then $y^*\in \mathrm{int}\,C^*$.

The following example describes some cones which we shall use in Sect. 6. Further examples can be found in, for instance, Berman et al. (1989), Berman and Plemmons (1994) and Krasnosel’skij et al. (1989).

### Example 2.2

(a): Let $X={\mathbb {R}}^n$. Then $C={\mathbb {R}}_+^n$ is a reproducing cone. The dual cone $C^*$ can be identified with *C* and $\mathrm{int}\,C^*=\mathrm{int}\,C=(0,\infty )^n$.
(b): Let $\Omega \subset {\mathbb {R}}^q$ be measurable, and $X=L^r(\Omega ,{\mathbb {R}})$, where $1\le r\le \infty$. Then $C=L^r(\Omega ,{\mathbb {R}}_+)$ is a reproducing cone, and, for $r<\infty$, the dual cone $C^*$ is given by $C^*=L^s(\Omega ,{\mathbb {R}}_+)$, where $1/r+1/s=1$. Note that $\mathrm{int}\,C^*\not =\emptyset$ if, and only if, $r=1$. If $r=1$, then $C^*=L^\infty (\Omega ,{\mathbb {R}}_+)$ and $h\in \mathrm{int}\,C^*$ if, and only if, ${{\,\mathrm{ess\,inf}\,}}\,h>0$.
(c): Let $X_1$ and $X_2$ be real Banach spaces, and let $C_1\subset X_1$ and $C_2\subset X_2$ be cones. Then $C_1\times C_2$ is a cone in $X_1\times X_2$. This cone is total (reproducing) if, and only if, both, $C_1$ and $C_2$ are total (reproducing). Moreover, $(C_1\times C_2)^*$ can be identified with $C_1^*\times C_2^*$. The interior of $(C_1\times C_2)^*$ is non-empty if, and only if, the interiors of each of $C_1^*$ and $C_2^*$ are non-empty. $\Diamond$

An operator $T\in {\mathcal {L}}(X)$ is said to be *positive* if $TC\subset C$, where $TC:=\{Tx:x\in C\}$, and in this case we write $T\ge 0$. Since

$$\begin{aligned} (T^*x^*)(x)=x^*(Tx)\quad \forall \, x\in X,\,\forall \, x^*\in X^*\,, \end{aligned}$$

it follows that if *T* is positive, then $T^*$ is positive.

We say that an operator $T\in {\mathcal {L}}(X)$ is exponentially stable if there exists $M\ge 1$ and $\mu \in (0,1)$ such that $\Vert T^t\Vert \le M\mu ^t$ for all $t\in {\mathbb {Z}}_+$, or equivalently, if the spectrum of *T* is contained in the open unit disc of the complex plane.

Let *Y* be a normed vector space. For $S\subset Y$, let ${\mathcal {F}}({\mathbb {Z}}_+,S)$ denote the set of all functions ${\mathbb {Z}}_+\rightarrow S$. For $u\in {\mathcal {F}}({\mathbb {Z}}_+,Y)$ and $t\in {\mathbb {Z}}_+$, we set

$$\begin{aligned} \Vert u\Vert _{\ell ^\infty (0,t)}:=\max \big \{\Vert u(\tau )\Vert :\tau \in {\mathbb {Z}}_+,\,\tau \le t\big \}\,. \end{aligned}$$

If $u\in {\mathcal {F}}({\mathbb {Z}}_+,Y)$ is bounded, then we define $\Vert u\Vert _{\ell ^\infty }:=\sup \{\Vert u(t)\Vert :t\in {\mathbb {Z}}_+\}$.

For the proof of the stability theorems in Sect. 5, we require an input-to-state stability result from control theory. To explain this result, consider the system

2.2 $$\begin{aligned} x^\nabla =Ax+bh(c^*x)+v,\quad x(0)=x^0\in X\,, \end{aligned}$$

where $x^\nabla$ is the left-shifted version of *x*, that is, $x^\nabla (t)=x(t+1)$, $A\in {\mathcal {L}}(X)$, $b\in X$ and $c^*\in X^*$, $h:{\mathbb {R}}\rightarrow {\mathbb {R}}$ is a continuous nonlinearity and $v\in {\mathcal {F}}({\mathbb {Z}}_+,X)$ is a forcing function (input, control). In control theory, systems of the form (2.2) are sometimes referred to as Lur’e systems. Obviously, (2.2) can be thought of as a feedback system obtained by application of the feedback law $\omega =h(y)$ to the linear controlled and observed system

2.3 $$\begin{aligned} x^\nabla =Ax+b\omega +v,\quad y=c^*x,\quad x(0)=x^0\,. \end{aligned}$$

The functions $\omega$ and *v* in (2.3) are interpreted as inputs (controls, disturbances, forcing functions) and *y* is called the output (measurement, observation) of (2.3). If $v=0$, then (2.3) reduces to

2.4 $$\begin{aligned} x^\nabla =Ax+b\omega ,\quad y=c^*x,\quad x(0)=x^0\,. \end{aligned}$$

Associated with (2.4) is the so-called transfer function

2.5 $$\begin{aligned} {{\mathbf {G}}}(z):=c^*_{\mathrm{c}}(zI-A_{\mathrm{c}})^{-1}b\,, \end{aligned}$$

where *z* is a complex variable. Here $A_{\mathrm{c}}$ and $c^*_{\mathrm{c}}$ denote the canonical complex extensions of *A* and $c^*$, respectively, to $X_{\mathrm{c}}$, the complexification of *X*; see, for example Deimling (1985).

We collect some properties of the discrete-time linear control system (2.4) and its transfer function (2.5). What follows is standard in control theory, but hard to find in one location in the infinite-dimensional setting. Suitable references include Staffans (2005), Chapter 12 and Coughlan (2007), Chapter 3 and Section 6.1. Applying the *Z*-transform (denoted by ${\mathcal {Z}}$) to (2.4), we obtain

$$\begin{aligned} ({\mathcal {Z}}y)(z)=zc^*_{\mathrm{c}}(zI-A_{\mathrm{c}})^{-1}x^0+{{\mathbf {G}}}(z)({\mathcal {Z}}\omega )(z)\,. \end{aligned}$$

The above identity shows that, in the frequency domain, the effect of the input on the linear dynamics is described by the product of the transfer function and the *Z*-transform of the input.

If *A* is exponentially stable, then we set

$$\begin{aligned} \Vert {{\mathbf {G}}}\Vert _{H^\infty }:=\sup _{|z|=1}|{{\mathbf {G}}}(z)|=\sup _{|z|\ge 1}|{{\mathbf {G}}}(z)|\,, \end{aligned}$$

where $H^\infty$ refers to the space of all bounded holomorphic functions defined on the complement of the closed unit disc. Denoting the output of (2.4) corresponding to the initial condition $x(0)=0$ by $y(\omega )$, we have that

$$\begin{aligned} \sup \big \{\Vert y(\omega )\Vert _{\ell ^2}:\Vert \omega \Vert _{\ell ^2}=1\big \}= \Vert {{\mathbf {G}}}\Vert _{H^\infty },\quad \text{ where }\,\,\, \Vert \omega \Vert _{\ell ^2}:=\Big (\sum _{t=0}^\infty |\omega (t)|^2\Big )^{1/2}\,, \end{aligned}$$

which provides an appealing interpretation of $\Vert {{\mathbf {G}}}\Vert _{H^\infty }$ in time-domain terms.

For later purposes, we note that, for every complex number $\zeta$,

2.6 $$\begin{aligned} c^*_{\mathrm{c}}(zI-(A_{\mathrm{c}}+\zeta bc^*_{\mathrm{c}}))^{-1}b=\frac{{{\mathbf {G}}}(z)}{1-\zeta {{\mathbf {G}}}(z)}\,. \end{aligned}$$

If *A* is exponentially stable, then the function on the right-hand side of (2.6) is meromorphic on the complement of the closed unit disc.

The following result is a special case of Gilmore et al. (2019), Corollary 3.3 and shall underpin our stability results in Sect. 5.

### Theorem 2.3

Consider the system (2.2), denote its solution by $x(\cdot \,;x^0,v)$, and assume that *h* is continuous and *A* is exponentially stable. If

$$\begin{aligned} \sup _{y\in {\mathbb {R}},\, y\not =0}|h(y)/y|<1/\Vert {{\mathbf {G}}}\Vert _{H^\infty }\,, \end{aligned}$$

where $1/\Vert {{\mathbf {G}}}\Vert _{H^\infty }:=\infty$ if ${{\mathbf {G}}}(z)\equiv 0$, then there exist $M\ge 1$, $\mu \in (0,1)$ and $N>0$ such that

2.7 $$\begin{aligned} \Vert x(t;x^0,v)\Vert \le M\mu ^t\Vert x^0\Vert +N\Vert v\Vert _{\ell ^\infty (0,t)} \,\,\forall \, t\in {\mathbb {Z}}_+,\,\forall \, x^0\in {\mathbb {R}}^n,\,\forall \, v\in {\mathcal {F}}({\mathbb {Z}}_+,X)\,. \end{aligned}$$

Recall that in a control theoretic setting *v* and *x* in (2.2) are called the input and state, respectively. If system (2.2) satisfies (2.7) (for some $M\ge 1$, $\mu \in (0,1)$ and $N>0$), then the zero equilibrium of the unforced ($v(t)\equiv 0$) system $x^\nabla =Ax+bh(c^*x)$ is said to be exponentially input-to-state stable (ISS). Frequently, for brevity, it is also said that (2.2) is exponentially ISS. Obviously, exponential ISS implies ISS, and the ISS concept is a standard stability concept in nonlinear control theory. It was defined by Sontag in the 1989 paper (Sontag 1989) in the context of general forced (or controlled) finite-dimensional nonlinear systems, and subsequently, a substantial Lyapunov theoretic ISS framework has been developed in the finite-dimensional setting, see, for example, Dashkovskiy et al. (2011), Jiang and Wang (2001), and Sontag (2006). ISS is a crucial concept as stability properties for nonlinear forced systems do not, in general, follow from stability properties of the *unforced* system; see, for example Teel and Hespanha (2004). Note that the converse to the above is true in the sense that (2.7) does imply that the zero equilibrium of the unforced system $x^\nabla =Ax+bh(c^*x)$ is globally exponentially stable (GES).

## Density-dependent population models with forcing

We consider the forced difference equation (1.1). Here $A\in {\mathcal {L}}(X)$ is positive, *b* and $c^*$ are non-zero elements in *C* and $C^*$, respectively, and $f:U\times {\mathbb {R}}_+\rightarrow {\mathbb {R}}_+$ is a continuous nonlinearity, where $U\subset {\mathbb {R}}$ is compact. The functions $u\in {\mathcal {F}}({\mathbb {Z}}_+,U)$ and $v\in {\mathcal {F}}({\mathbb {Z}}_+,C)$ are forcing terms, and we shall always assume that the initial condition belongs to the positive cone, $x^0 \in C$. Recall that (1.1) can be thought of as the feedback interconnection of $\omega =f(u,y)$ and the linear system (2.3).

Typical scenarios for *f* include:

- $f(w,y)=g(wy)y$, where $g:(0,\infty )\rightarrow {\mathbb {R}}_+$ is continuous and such that $\lim _{y\rightarrow 0}g(y)y$ exists and is finite;
- $f(w,y)=g(wy)$, where $g:{\mathbb {R}}_+\rightarrow {\mathbb {R}}_+$ is continuous;
- $f(w,y)=wg(y)$, where $g:{\mathbb {R}}_+\rightarrow {\mathbb {R}}_+$ is continuous.

In each of the above cases, *U* is a compact subset of ${\mathbb {R}}_+$. We point out that there are interesting and relevant examples of nonlinearities which do not fall into any of the above three scenarios, one such example is given by $f(w,y)=y/(w+y^{\alpha })$, where $\alpha >0$ and $\alpha \not =1$.^1^

We impose the following positivity and stability assumptions on the linear system determined by *A*, *b* and $c^*$.

(L1): $A\in {\mathcal {L}}(X)$ is positive and exponentially stable.
(L2): $$b\in C$$, $$c^*\in C^*$$, $$b\not =0$$ and $$c^*\not =0$$.

Further assumptions on *f* will be specified in Sects. 4 and 5.

The positivity conditions in (L1) and (L2), together with the assumption that the values of *f* are non-negative, imply that, for all $x^0\in C$, $u\in {\mathcal {F}}({\mathbb {Z}}_+,U)$ and $v\in {\mathcal {F}}({\mathbb {Z}}_+,C)$, the solution *x* of (1.1) remains in the cone *C*.

Our next result introduces a key quantity, denoted *p*, associated with (1.1), and records some elementary consequences of (L1) and (L2).

### Proposition 3.1

Assume that (L1) and (L2) are satisfied, and let ${{\mathbf {G}}}$ be given by (2.5). The following statements hold.

(a): $$\Vert {{\mathbf {G}}}\Vert _{H^\infty }={{\mathbf {G}}}(1)\ge 0$$ and
  3.1 $$\begin{aligned} p:=\frac{1}{{{\mathbf {G}}}(1)}\in (0,\infty ],\quad \text{ where } \,\,p:=\infty \quad \text{ if } {{\mathbf {G}}}(1)=0. \end{aligned}$$
(b): The operator $A+\lambda bc^*$ is exponentially stable for all $\lambda \in [0,p)$.
(c): If $p<\infty$, then 1 is an eigenvalue of $A_{\mathrm{c}}+pbc_{\mathrm{c}}^*$, in particular, $A+pbc^*$ is not exponentially stable.

The number *p* in Proposition 3.1, which will play a crucial role throughout, is equal to the stability radius of the linear system specified by *A*, with perturbation structure *b* and $c^*$, as captured by statements (b) and (c) of Proposition 3.1. That is, $p = \min \vert k \vert$, where the minimum is taken over all *k* such that $A+kbc^*$ is *not* exponentially stable.

The analysis of boundedness, stability and persistence properties of (1.1) relies on the interplay between *p*, capturing stability of the linear component, and the nonlinear term *f*, combined with suitable positivity properties. The interplay between *p* and *f* is described mathematically by our nonlinear assumptions (N1), (N2) and (N3) in Sects. 4 and 5.

A biological interpretation of *p* depends on the specific context. For instance, recall that, in our usual setting (1.1) models a single, stage-structured population, subject to forcing. The right hand side of (1.1) captures the life-cycle over one time-step, including growth/transitions between stage-classes, and recruitment into the population. The form of (1.1) contains both linear and non-linear terms, corresponding to processes which are assumed to be density independent and density dependent, respectively. Following the interpretation given in Eager (2016), Rebarber et al. (2012), Townley et al. (2012), for a population distribution *x*, the term *Ax* describes survival and growth, and $bf(w,c^*x)$ models density-dependent recruitment (combining fecundity of reproductive individuals and survival of offspring). Here offspring are distributed into the population according to *b*; and there are (at least) two interpretations of $f(w,c^* x)$:

(i) $c^*x$ is the number of reproductive members of the population, and *f* is the density-dependent recruitment, or;
(ii) the number of offspring is $c^*x$ and multiplied by $f(w,c^*x)/c^*x$, the density dependent per-capita survival or establishment probability, yielding total recruitment. Clearly, for this interpretation to be biologically meaningful $y \mapsto f(w,y)/y$ must take values in [0, 1].

The setting (ii) is adopted in Eager (2016), and more biological context is given there. Indeed, we quote (Eager 2016, p. 43): “*p* is simply the establishment probability that would cause stasis in a density-independent setting [meaning $f(y) = p y$ for all *y*].” Further, it is noted in Eager (2016), p. 44 that *p* is also equal to the reciprocal of the inherent net reproductive number of $A + bc^*$ (in the sense of Cushing (1998), p. 7), see Eager (2016), p. 44 for more details.

## Boundedness and persistence

In this section we address the first two properties of boundedness and persistence. Assume that (L1) and (L2) hold, and let $U\subset {\mathbb {R}}$ be compact. Throughout, *U* will be the range of permitted values for the scalar forcing term *u*. In the special cases we have in mind, $U\subset {\mathbb {R}}_+$ with $0\not \in U$ (see examples further below), but this is not required in the general theory we will develop.

For the rest of the paper, for given $x^0 \in C$, $u \in {\mathcal {F}}({\mathbb {Z}}_+,U)$ and $v \in {\mathcal {F}}({\mathbb {Z}}_+, C)$ we let $x = x(\cdot \,;x^0,u,v)$ denote the solution of (1.1), which is obviously unique. As a matter of convenience, we set

$$\begin{aligned} C_\rho :=C\cap {\mathbb {B}}(0,\rho ),\quad \text{ where } \rho >0\,. \end{aligned}$$

Let *Y* be a real normed space and $T\in {\mathcal {L}}(X,Y)$. We say that (1.1) is *ultimately semi-globallyT*-*persistent* if, for every bounded closed set $\Gamma \subset C$ with $0\not \in \Gamma$ and every $\rho >0$, there exist $\tau \in {\mathbb {Z}}_+$ and $\eta >0$ such that, for all $x^0\in \Gamma$, $u\in {\mathcal {F}}({\mathbb {Z}}_+,U)$ and all $v\in {\mathcal {F}}({\mathbb {Z}}_+,C_\rho )$,

$$\begin{aligned} \Vert Tx(t+\tau ;x^0,u,v)\Vert \ge \eta \quad \forall \, t\in {\mathbb {Z}}_+\,. \end{aligned}$$

Note that this persistence property is “semi-global” in contrast to the global persistency concepts for unforced systems considered in Smith and Thieme (2011, 2013). On the other hand, the above concept has stronger uniformity properties than those in Smith and Thieme (2011, 2013): the same $\tau$ “works” for all $x^0\in \Gamma$, $u\in {\mathcal {F}}({\mathbb {Z}}_+,U)$ and all $v\in {\mathcal {F}}({\mathbb {Z}}_+,C_\rho )$. The relevance of persistence in the context of population dynamics is obvious: its absence is equivalent to extinction. For all practical purposes the semi-global nature of the above persistency concept is sufficient: for any given application context, there exists a bounded closed set $\Gamma \subset C$ such that every practically relevant initial condition will belong to $\Gamma$. In the special case wherein $Y=X$ and $T=I$, we use the term “persistent” rather than “*I*-persistent”. If, in the above definition, $\tau =0$, then we simply say that (1.1) is *semi-globallyT*-*persistent*.

We remark that if, for every $u\in U$, the function $y\mapsto f(u,y)$ is non-decreasing, then, for $z^*\in C^*$, the system (1.1) is ultimately semi-globally $z^*$-persistent if, and only if, for every bounded closed set $\Gamma \subset C$, $0\not \in \Gamma$, there exist $\tau \in {\mathbb {Z}}_+$ and $\eta >0$ such that $z^*(x(t+\tau ;x^0,u,0))\ge \eta$ for all $x^0\in \Gamma$, $u\in {\mathcal {F}}({\mathbb {Z}}_+,U)$ and all $t\in {\mathbb {Z}}_+$, that is, the forcing *v* is irrelevant for persistency in this scenario. Moreover, if additionally the norm on *X* is monotone, then (1.1) is ultimately semi-globally persistent if, and only if, for every bounded closed set $\Gamma \subset C$, $0\not \in \Gamma$, there exist $\tau \in {\mathbb {Z}}_+$ and $\eta >0$ such that $\Vert x(t+\tau ;x^0,u,0)\Vert \ge \eta$ for all $x^0\in \Gamma$, $u\in {\mathcal {F}}({\mathbb {Z}}_+,U)$ and all $t\in {\mathbb {Z}}_+$. In summary, under certain additional assumptions on *f* and the positive cone *C*, we can decouple persistence of (1.1) from the additive forcing *v*.

The following result gives a necessary condition for persistence.

### Proposition 4.1

Assume that (L1) and (L2) hold and $f(w,0)=0$ for all $w\in U$. If (1.1) is ultimately semi-globally persistent, then $c^*(I-A)^{-1}x>0$ for all $x\in C$, $x\not =0$. In particular, ${{\mathbf {G}}}(1)=c^*(I-A)^{-1}b>0$, or, equivalently, $p<\infty$, where *p* is given by (3.1).

Next we introduce suitable assumptions on *f* which will enable us to prove boundedness and persistence results for the system (1.1).

(N1): $U\subset {\mathbb {R}}$ is compact, $f:U\times {\mathbb {R}}_+\rightarrow {\mathbb {R}}_+$ is continuous, $f(w,y)>0$ for all $w\in U$ and $y>0$ and
  4.1 $$\begin{aligned} \limsup _{y\rightarrow \infty }\frac{\max _{w\in U}f(w,y)}{y}<p\,, \end{aligned}$$
  where *p* is given by (3.1).
(N2):  (N1) holds and
  4.2 $$\begin{aligned} \liminf _{y\rightarrow 0}\frac{\min _{w\in U}f(w,y)}{y}>p\,. \end{aligned}$$

### Remark 4.2

We provide some interpretations of the assumptions (N1) and (N2) in the biological context for (1.1) described at the end of Sect. 3. In the setting (ii), (N1) means that, at large population abundance, the per-capita survival probability *f*(*w*, *y*) / *y* is less than *p*, the level which results in population stasis. We require that the inequality holds in the “worst case” across all possible control actions *w*, hence the maximum over *w* in (4.1). In essence, (N1) prevents populations from becoming unboundedly large, as there is a sufficiently diminishing return in recruitment at very high population abundance.

Whereas (N1) pertains solely to large populations, (N2) pertains to small populations as well. Specifically the latter requires that, at low population abundance, the per-capita survival probability *f*(*w*, *y*) / *y* is greater than *p*, again with the inequality holding in the “worst case” across all possible control actions, hence the minimum over *w* in (4.2). Consequently, at low population abundances, we expect to see population growth. In particular, we do not have an Allee effect see, for example, Taylor and Hastings (2005).

Note that neither (N1) nor (N2) place monotonicity constraints on *f*. $\Diamond$

The following result provides classes of examples for which conditions (N1) or (N2) are satisfied.

### Proposition 4.3

Let $U\subset {\mathbb {R}}_+$ be compact, $0\not \in U$ and set $u^-:=\min U$ and $u^+:=\max U$, in which case $0<u^-\le u^+<\infty$.

(a): Let $g:(0,\infty )\rightarrow (0,\infty )$ be continuous and such that $f_0:=\lim _{y\rightarrow 0}g(y)y$ exists and is finite, and define $f:U\times {\mathbb {R}}_+\rightarrow {\mathbb {R}}_+$ by
  $$\begin{aligned} f(w,y)=\left\{ \begin{array}{l l} g(wy)y,&{}\quad \text {for }w\in U \text { and }y>0 \\ f_0/w, &{}\quad \text {for }w\in U\text { and }y=0.\end{array}\right. \end{aligned}$$
  If $\limsup _{y\rightarrow \infty }g(y)<p$, then *f* satisfies condition (N1). If additionally, $\liminf _{y\rightarrow 0}g(y)>p$, then *f* satisfies (N2).
(b): Let $g:{\mathbb {R}}_+\rightarrow {\mathbb {R}}_+$ be continuous and such that $g(y)>0$ for all $y>0$, and define $f:U\times {\mathbb {R}}_+\rightarrow {\mathbb {R}}_+$ by $f(w,y):=g(wy)$ or by $f(w,y):=wg(y)$ for all $w\in U$ and $y\in {\mathbb {R}}_+$. If $\limsup _{y\rightarrow \infty }(g(y)/y)<p/u^+$, then *f* satisfies (N1). If additionally, $\liminf _{y\rightarrow 0}g(y)/y>p/u^-$, then *f* satisfies (N2).

The next result shows that (N1) and (N2), in combination with (L1) and (L2) and a suitable “strict” positivity assumption (see (P1) below), are sufficient to establish semi-global boundedness and persistence properties of (1.1). We remark that assumption (N2) will have to be somewhat strengthened for the stability results in Sect. 5.

### Theorem 4.4

Consider the initial-value problem (1.1) and let *p* be given by (3.1). Assume that (L1) and (L2) hold, and let $\rho >0$. The following statements hold.

(a): If (N1) is satisfied and $\Gamma \subset C$ is bounded, then there exists $\gamma >0$ such that, for all $x^0\in \Gamma$, $u\in {\mathcal {F}}({\mathbb {Z}}_+,U)$ and $v\in {\mathcal {F}}({\mathbb {Z}}_+,C_\rho )$,
  $$\begin{aligned} \Vert x(t;x^0,u,v)\Vert \le \gamma \quad \forall \, t\in {\mathbb {Z}}_+\,. \end{aligned}$$
(b): Assume that (N2) and the following additional condition hold.

(P1): There exists $\tau \in {\mathbb {Z}}_+$ such that $c^*(A+bc^*)^\tau \in \mathrm{int}\,C^*$.

If $\Gamma \subset C$ is bounded, closed and such that $0\not \in \Gamma$, then there exist $\delta >0$ and $\eta >0$ such that, for all $x^0\in \Gamma$, $u\in {\mathcal {F}}({\mathbb {Z}}_+,U)$ and $v\in {\mathcal {F}}({\mathbb {Z}}_+,C_\rho )$,

$$\begin{aligned} \Vert x(t;x^0,u,v)\Vert \ge \delta \quad \text{ and }\quad c^*x(t+\tau ;x^0,u,v)\ge \eta \qquad \forall \, t\in {\mathbb {Z}}_+\,. \end{aligned}$$

In particular, (1.1) is semi-globally persistent and ultimately semi-globally $c^*$-persistent.

Statement (a) of Lemma 4.5 below shows that (P1) is implied by each of the following “positivity” conditions.

(P2): There exists $\tau \in {\mathbb {Z}}$ such that $c^*\sum _{t=0}^\tau (A+bc^*)^t\in \mathrm{int}\,C^*$ and $c^*b>0$.
(P3): There exists $\tau \in {\mathbb {N}}$ such that $[(A+bc^*)^*]^\tau$ is strictly positive, that is, $[(A+bc^*)^*]^\tau x^*\in \mathrm{int}\,C^*$ for all non-zero $x^*\in C^*$.

The simple example given by $X={\mathbb {R}}^3$, $C={\mathbb {R}}_+^3$ and

$$\begin{aligned} A=\begin{pmatrix}\alpha _1 &{} 0 &{} 0 \\ 0 &{} \alpha _2 &{} \alpha _3\\ 0 &{} 0 &{} 0\end{pmatrix},\quad b=\begin{pmatrix} 0 \\ 0 \\ \beta \end{pmatrix},\quad c^*=\left( \begin{matrix} \gamma _1&\gamma _2&0 \end{matrix} \right) ,\quad \text{ where } \alpha _i,\beta ,\gamma _i>0, \end{aligned}$$

shows that (P1) does not imply (P2) or (P3).^2^

### Lemma 4.5

Assume that (L1) and (L2) are satisfied. The following statements hold.

(a): If (P2) or (P3) holds, then (P1) is satisfied.
(b): Assume that $X={\mathbb {R}}^n$ and $C={\mathbb {R}}^n_+$. If $A+bc^*$ is irreducible and $c^*b>0$, then (P1), (P2) and (P3) hold.
(c): If (P3) holds, then, for every $\lambda >0$, the operator $[(A+\lambda bc^*)^*]^\tau$ is strictly positive, that is, $[(A+\lambda bc^*)^*]^\tau x^*\in \mathrm{int}\,C^*$ for every non-zero $x^*\in C^*$.

We provide some comments relating the above result to the persistency theory developed in Franco et al. (2017), and on the assumption (P1).

### Remark 4.6

(a): The theory developed in Franco et al. (2017) is restricted to the situation wherein $X = {\mathbb {R}}^n$ and $C = {\mathbb {R}}^n_+$, whilst here we consider any total cone satisfying $\mathrm{int}\,C^* \ne \emptyset$, and the state space *X* may be infinite dimensional. Theorem 4.4 extends (Franco et al. 2017, Theorem 4.2) in several directions: as already mentioned, the underlying linear system is allowed to be infinite-dimensional; further, the positivity condition (P1) is less restrictive than the primitivity of $A+bc^*$, which is imposed in Franco et al. (2017), Townley et al. (2012), and, finally; the structure of the forced system (1.1) is more general than that considered in Franco et al. (2017).
(b): Another difference between the present work and Franco et al. (2017) is the argumentation: a key point in both works is the formulation of assumptions ensuring the existence of $z^* \in \mathrm{int}\,C^*$ such that
  4.3 $$\begin{aligned} z^*(A + pbc^*) = z^*\,, \end{aligned}$$
  that is, guaranteeing the existence of a (strictly) positive left eigenvector of $A +pbc^*$ corresponding to the eigenvalue 1. In the case wherein $X={\mathbb {R}}^n$ and $C={\mathbb {R}}_+^n$, if (P3) holds (or, equivalently, if $A + pbc^*$ is primitive), then Perron–Frobenius theory (Berman and Plemmons 1994; Meyer 2000) guarantees the existence of such a (strictly) positive left eigenvector and this is exploited by the authors of the papers (Franco et al. 2017; Townley et al. 2012) in which (P3), rather than (P1), plays a key role. Similarly, in the infinite-dimensional case, (P3) and a variant of the Krein–Rutman theorem (Deimling 1985, Theorem 19.3) can be used to establish the existence of a $z^*\in \mathrm{int}\,C^*$ satisfying (4.3) provided that *A* is compact. However, assumption (P1) is sufficient to explicitly construct, from first principles, a functional $z^*\in \mathrm{int}\,C^*$ which satisfies (4.3) without assuming compactness of *A*, see Lemma A.1 for details. The upshot is that, under weaker hypotheses than in Franco et al. (2017), Townley et al. (2012) we obtain stronger conclusions (in the sense that they cover the infinite-dimensional case).
(c): The persistence properties guaranteed by Theorem 4.4 overlap with persistency concepts (for systems without forcing) introduced in Freedman and So (1989), Smith and Thieme (2011, 2013), Wen et al. (2016). In particular, it follows immediately from statement (b) of Theorem 4.4 that the unforced system (1.1) is strongly $\Vert \cdot \Vert$-persistent and strongly $c^*$-persistent, respectively, in the sense of Smith and Thieme (2011), Definition 3.1.
(d): Note that (P1) implies that $\mathrm{int}\,C^*\not =\emptyset$. The latter is certainly satisfied in the finite-dimensional case, but may fail to hold for many infinite-dimensional spaces, see Example 2.2. However, the dual cone of $L^1(\Omega , {\mathbb {R}}_+)$, where $\Omega \subset {\mathbb {R}}^n$ is measurable, is nonempty. As discussed in Sect. 6.2, $L^1(\Omega , {\mathbb {R}}_+)$ is a natural state space for numerous biologically meaningful infinite-dimensional models. Finally, we point out that the key positivity assumption imposed in Rebarber et al. (2012), Theorem 3.3 is the condition $c^*\in \mathrm{int}\,C^*$, i.e., (P1) with $\tau =0$. $\Diamond$

## Stability

Having established boundedness and persistence results in Sect. 4 (under suitable assumptions), we are now interested in conditions which will guarantee the existence of a “stable” non-zero equilibrium: the stability notion used here takes into account the forcing terms *u* and *v* and is reminiscent of the input-to-state stability (ISS) concept from nonlinear control theory, introduced in Sontag (1989); see more recently Dashkovskiy et al. (2011), Karafyllis and Jiang (2011) and Sontag (2006). Indeed, the ISS result Theorem 2.3 is a crucial ingredient in what follows. As has been mentioned already, to derive useful stability results, we need to somewhat strengthen the assumption (N2) on *f*.

In the following, let *p* be the constant given by (3.1) and let $u^{\mathrm{e}}\in U$, where $U\subset {\mathbb {R}}$ is compact. The number $u^{\mathrm{e}}$ will play the role of a target or nominal value for the control variable *u*.

The nonlinearity *f* appearing in (1.1) is assumed to satisfy the following conditions.

(N3): Condition (N2) holds,
  5.1 $$\begin{aligned} |f(u^{\mathrm{e}},y)-f(u^{\mathrm{e}},y^{\mathrm{e}})|=|f(u^{\mathrm{e}},y)-py^{\mathrm{e}}| <p|y-y^{\mathrm{e}}|\quad \forall \, y>0,\,y\not =y^{\mathrm{e}}\,, \end{aligned}$$
  where $y^{\mathrm{e}}$ is the unique positive number such that $f(u^{\mathrm{e}},y^{\mathrm{e}}) =py^{\mathrm{e}}$, and
  5.2 $$\begin{aligned} \limsup _{y\rightarrow y^{\mathrm{e}}}\frac{|f(u^{\mathrm{e}},y)-f(u^{\mathrm{e}},y^{\mathrm{e}})|}{|y-y^{\mathrm{e}}|} =\limsup _{y\rightarrow y^{\mathrm{e}}}\frac{|f(u^{\mathrm{e}},y)-py^{\mathrm{e}}|}{|y-y^{\mathrm{e}}|}<p\,. \end{aligned}$$

The existence of $y^{\mathrm{e}}>0$ such that $f(u^{\mathrm{e}},y^{\mathrm{e}})=py^{\mathrm{e}}$ follows from the continuity of *f*, (4.1) and (4.2), whilst uniqueness of $y^{\mathrm{e}}$ is a consequence of (5.1).

**Fig. 1:**
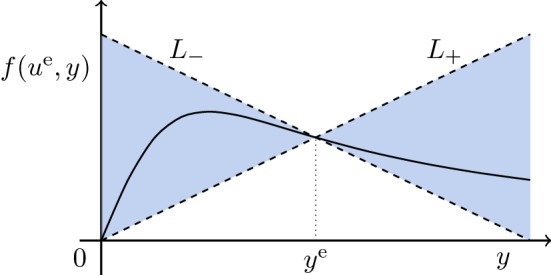
Illustration of the sector condition (5.1)

Note that (5.1) is a sector condition and means that the graph *F* of $y\mapsto f(u^{\mathrm{e}},y)$ is “sandwiched” between the lines $L_+=\{(y,py):y\ge 0\}$ and $L_-=\{(y,-py + 2py^{\mathrm{e}}):y\ge 0\}$, see Fig. 1 for an illustration. Obviously, the only points the graph *F* has in common with $L_+$ or $L_-$ are (0, 0) and $(y^{\mathrm{e}}, py^{\mathrm{e}})$. Condition (5.2) implies that the intersections of *F* with $L_+$ and $L_-$ at the point $(y^{\mathrm{e}}, py^{\mathrm{e}})$ is non-tangential, whilst (4.2) ensures that *F* is non-tangential to $L_+$ at (0, 0). Finally, it follows from the continuity of *f* and (4.1), (4.2), (5.1) and (5.2) that, for every $\eta >0$, there exists $q\in (0,p)$ such that

5.3 $$\begin{aligned} |f(u^{\mathrm{e}},y)-f(u^{\mathrm{e}},y^{\mathrm{e}})|=|f(u^{\mathrm{e}},y)-py^{\mathrm{e}}| \le q|y-y^{\mathrm{e}}|\quad \forall \, y\ge \eta ,\,y\not =y^{\mathrm{e}}\,. \end{aligned}$$

The following lemma shows the existence of a unique non-zero equilibrium of the Lur’e system $x^\nabla =Ax+bf(u^{\mathrm{e}},c^*x)$.

### Lemma 5.1

Assume that (L1),  (L2) and (N3) hold, and set

5.4 $$\begin{aligned} x^{\mathrm{e}}:=(I-A)^{-1}bpy^{\mathrm{e}}\,, \end{aligned}$$

where *p* is given by (3.1), and $y^{\mathrm{e}}$ is as in (N3). Then $x^{\mathrm{e}}>0$, $c^*x^{\mathrm{e}}=y^{\mathrm{e}}$, and $x^{\mathrm{e}}$ is the unique non-zero equilibrium of (1.1) with $u(t)\equiv u^{\mathrm{e}}$ and $v(t)\equiv 0$.

Furthermore, if (P3) holds, then $x^*(x^{\mathrm{e}})>0$ for all non-zero $x^*\in C^*$.

We are now in the position to state a stability theorem relating to the non-zero equilibrium of system (1.1).

### Theorem 5.2

Let $U\subset {\mathbb {R}}$ be compact and $u^{\mathrm{e}}\in U$. Assume that (1.1) is ultimately semi-globally $c^*$-persistent and (L1), (L2) and (N3) hold. Furthermore, let $x^{\mathrm{e}}>0$ be given by (5.4). The following statements hold.

(a): Let $\Gamma \subset C$ be bounded, closed and such that $0\not \in \Gamma$, and let $\rho >0$. Then there exist $M\ge 1$, $\mu \in (0,1)$ and $N>0$ such that, for all $x^0\in \Gamma$, $u\in {\mathcal {F}}({\mathbb {Z}}_+,U)$ and $v\in {\mathcal {F}}({\mathbb {Z}}_+,C_\rho )$,
  5.5 $$\begin{aligned} \Vert x(t;x^0,u,v)-x^{\mathrm{e}}\Vert \le M\mu ^t\Vert x^0-x^{\mathrm{e}}\Vert +N(\beta (u,t)+ \Vert v\Vert _{\ell ^\infty (0,t)})\quad \forall \, t\in {\mathbb {Z}}_+, \end{aligned}$$
  with
  $$\begin{aligned} \beta (u,t):=\max \{|f(u^{\mathrm{e}},y)-f(u(s),y)|: s=0,1,\ldots ,t,\,\,\,0\le y\le \gamma \Vert c^*\Vert \}, \end{aligned}$$
  where $\gamma$ is the constant from statement (a) of Theorem 4.4.
(b): For every $x^0\in C$, $x^0\not =0$, every $u\in {\mathcal {F}}({\mathbb {Z}}_+,U)$ and every $v\in {\mathcal {F}}({\mathbb {Z}}_+,C)$ such that $u(t)\rightarrow u^{\mathrm{e}}$ and $v(t)\rightarrow 0$ as $t\rightarrow \infty$, we have that $x(t;x^0,u,v)\rightarrow x^{\mathrm{e}}$ as $t\rightarrow \infty$.

Note that if $(u_k)_{k\in {\mathbb {N}}}$ is a sequence in ${\mathcal {F}}({\mathbb {Z}}_+,U)$ such that $u_k$ converges to (the constant function) $u^{\mathrm{e}}$ in the sup-norm as $k\rightarrow \infty$, then $\lim _{k\rightarrow \infty }\big [\sup _{t\in {\mathbb {Z}}_+}\beta (u_k,t)\big ]=0$.

The following corollary is an immediate consequence of Theorem 4.4, statement (a) of Lemma 4.5, and Theorem 5.2.

### Corollary 5.3

Assume that (L1), (L2), (N3) hold, and at least one of the conditions (P1)–(P3) is satisfied. Then statements (a) and (b) of Theorem 5.2 hold.

The following illustrative example discusses stability properties of a simple model for which (P1) holds, but (P3) does not.

### Example 5.4

Consider the system (1.1) with

$$\begin{aligned} A=\begin{pmatrix}1/2 &{} 1/2 &{} a\\ 1/2 &{} 0 &{} 0\\ 0 &{} 0 &{} 0\end{pmatrix},\quad b=\begin{pmatrix} 0 \\ 0 \\ 1\end{pmatrix},\quad c^*=\begin{pmatrix} 0&1&1\end{pmatrix},\quad \text{ where } a\ge 0, \end{aligned}$$

and with the nonlinearity $f:U\times {\mathbb {R}}_+\rightarrow {\mathbb {R}}_+,\, (w,y)\mapsto 6y/(5+wy)$, where $U\subset {\mathbb {R}}_+$ is compact and such that $0\not \in U$ and $1\in U$.

The eigenvalues of *A* are independent of *a* and are given by 0 and $(1\pm \sqrt{5})/4$. Consequently, *A* is exponentially stable for all $a\ge 0$. In particular, (L1) and (L2) hold for all $a\ge 0$. Condition (P3) is satisfied if $a>0$. For $a=0$, (P3) is not satisfied, but (P1) holds. Moreover, we have that ${{\mathbf {G}}}(1)=1+2a$, showing that, with $u^{\mathrm{e}}=1$ and $y^{\mathrm{e}}=1+12a$, condition (N3) holds for every $a\ge 0$. Hence, the conclusions of Corollary 5.3 apply with

$$\begin{aligned} x^{\mathrm{e}}=\frac{1+12a}{1+2a}\begin{pmatrix} 4a \\ 2a \\ 1\end{pmatrix}\,. \end{aligned}$$

We now discuss the case wherein $a=0$. This case is somewhat degenerate because, for $a=0$, the components $x_1$ and $x_2$ are decoupled from $x_3$ (note the $A+bc^*$ is reducible) and the dynamics of the $(x_1,x_2)$-subsystem are linear and exponentially stable (for $v=0$). It is therefore clear (without using Corollary 5.3) that the first two components of $x^{\mathrm{e}}$ are equal to 0 and the third component $x^{\mathrm{e}}_3$ of $x^{\mathrm{e}}$ must be the unique positive fixed point of *f*, namely $x^{\mathrm{e}}_3=1$. It is interesting that Corollary 5.3 applies to this degenerate situation which is not covered by the results in Franco et al. (2017); Townley et al. (2012). $\Diamond$

In the following, we compare Theorem 5.2 and Corollary 5.3 with other stability results available in the literature.

### Remark 5.5

The papers Rebarber et al. (2012) and Smith and Thieme (2013) consider the uncontrolled version of system (1.1), both in an infinite-dimensional setting. We note that Theorem 5.2 and Corollary 5.3 are considerably more general than the theory developed in Rebarber et al. (2012), even in the unforced case and, in particular, contain (Rebarber et al. 2012, Theorem 3.3) as a special case. A comparison of Theorem 5.2 and Corollary 5.3 with results in Smith and Thieme (2013) (see Smith and Thieme (2013), part (b) of Theorem 5.8, part (c) of Theorem 7.1) is more difficult: whilst there is some overlap, the assumptions imposed and the methods used are quite different and certainly neither set of results contains the other, see also Example 6.2 in this context. $\Diamond$

In the special case that *f* is given by $f(w,y)=g(wy)y$, statement (b) of Theorem 5.2 can be strengthened.

### Corollary 5.6

Let $g:(0,\infty )\rightarrow (0,\infty )$ be continuous and such that the limit $\lim _{y\rightarrow 0}g(y)y$ exists,

5.6 $$\begin{aligned} \liminf _{y\rightarrow 0}g(y)>p\quad \text{ and }\quad \limsup _{y\rightarrow \infty }g(y)<p\,. \end{aligned}$$

Let $u^{\mathrm{e}}>0$ and assume that

5.7 $$\begin{aligned} |g(u^{\mathrm{e}}y)y-g(u^{\mathrm{e}}y^{\mathrm{e}})y^{\mathrm{e}}|=|g(u^{\mathrm{e}}y)y-py^{\mathrm{e}}| <p|y-y^{\mathrm{e}}|\quad \forall \, y>0,\,y\not =y^{\mathrm{e}}\,, \end{aligned}$$

where $y^{\mathrm{e}}$ is the unique positive number such that $g(u^{\mathrm{e}}y^{\mathrm{e}}) =p$, and

5.8 $$\begin{aligned} \limsup _{y\rightarrow y^{\mathrm{e}}}\frac{|g(u^{\mathrm{e}}y)y-g(u^{\mathrm{e}}y^{\mathrm{e}})y^{\mathrm{e}}|}{|y-y^{\mathrm{e}}|}=\limsup _{y\rightarrow y^{\mathrm{e}}}\frac{|g(u^{\mathrm{e}}y)y-py^{\mathrm{e}}|}{|y-y^{\mathrm{e}}|}<p\,. \end{aligned}$$

Furthermore, assume that the Lur’e system

5.9 $$\begin{aligned} x^\nabla =Ax+bg(uc^*x)c^*x+v,\quad x(0)=x^0\in C\,, \end{aligned}$$

is ultimately semi-globally $c^*$-persistent, (L1) and (L2) hold, and let $x^{\mathrm{e}}>0$ be given by (5.4). Then, for every $x^0\in C$, $x^0\not =0$, every $u^\infty >0$, every $u\in {\mathcal {F}}({\mathbb {Z}}_+,(0,\infty ))$ and every $v\in {\mathcal {F}}({\mathbb {Z}}_+,C)$ such that $u(t)\rightarrow u^\infty$ and $v(t)\rightarrow 0$ as $t\rightarrow \infty$, the solution $x(t;x^0,u,v)$ of (5.9) converges to the limit $(u^{\mathrm{e}}/{u^\infty })x^{\mathrm{e}}$ as $t\rightarrow \infty$.

## Examples

In this section, we apply the theory developed in the previous sections to a range of examples from population dynamics. Recall that our main results are Theorem 4.4 and Corollary 5.3 which ensure boundedness and persistence, and stability, respectively. We illustrate situations wherein our main assumptions: linear (L1) and (L2), nonlinear (N1)–(N3) and, positive (P1)–(P3) are satisfied. We present two finite-dimensional examples and then proceed to exploit the full power of our results by applying the abstract theory to a class of nonlinear integral projection models.

### Finite-dimensional examples

We first analyse a system which provides a simple model for the dynamics of a population with a refuge.

#### Example 6.1

Consider the following model

6.1 $$\begin{aligned} \left. \begin{aligned} x_1(t+1)&= (1-\varepsilon ) g(u(t) x_1(t))+\varepsilon ' (1-\mu ) x_2(t) + v_1(t),&x_1(0)&= x_1^0,\\ x_2(t+1)&=(1-\varepsilon ') (1-\mu ) x_2(t) +\varepsilon g(u(t) x_1(t))+ v_2(t),&x_2(0)&= x_2^0, \end{aligned} \right\} \quad \forall \, t \in {\mathbb {Z}}_+\,,\nonumber \\ \end{aligned}$$

where $x_1(t)$ and $x_2(t)$ denote the density of an active population and its refuge at time-step $t\in {\mathbb {Z}}_+$, respectively. Here the parameters $\varepsilon ,\varepsilon '\in (0,1)$ are the respective dispersal rates into and out of the refuge populations; the parameter $\mu \in [0,1]$ measures the attrition in the refuge; the nonlinearity *g* describes density-dependent reproduction of the active population; the forcing $u(t) \in U:=[u^-,u^+]\subseteq (0,\infty )$ models the effect of demographic fluctuations affecting recruitment, and; the terms $v_1(t), v_2(t) \in {\mathbb {R}}_+$ correspond to immigration. The model (6.1) was proposed in Newman et al. (2002), without demographic fluctuations and immigration, as the simplest possible model of an active population coupled to a refuge.

A simplified version of model (6.1) assuming symmetric dispersal, without immigration, neglecting attrition in the refuge and neglecting demographic fluctuations, that is, with $\varepsilon =\varepsilon '$, $\mu =0$, $u= 1$, and $v_1 = v_2 = 0$, has been studied in Chow et al. (2018), Newman et al. (2002). In Newman et al. (2002), the simplified model was studied numerically for two nonlinearities known to be able to produce complex dynamics, namely $g(y)=\lambda ye^{-y}$ and $g(y)=\lambda y(1-y)$ for fixed $\lambda >0$. The authors of Chow et al. (2018) studied the simplified model analytically with the choice of function $g(y)=\lambda y/(1+k y)$, which is a Beverton–Holt type nonlinearity, where $k>0$ is a parameter. Observe that for each function *g* given, $g'(0) = \lambda$ (the derivative at zero being a right derivative), and so $\lambda$ is the linearised population growth-rate at zero. The authors of Chow et al. (2018) prove persistence and global stability results using specific properties of the Beverton–Holt function, namely that it is strictly increasing and concave. Such an approach would not apply to the unimodal functions originally considered in Newman et al. (2002). The authors of Newman et al. (2002) noted the relevance of studying the more general model, indeed, we quote (Newman et al. 2002, p. 123): “These [the simpler] choices are made purely on the grounds of simplicity, and the investigation of non-zero attrition and asymmetric dispersal are certainly worthy of future study.”

We note that (6.1) is a special case of the system (1.1) with $X = {\mathbb {R}}^2$, $C = {\mathbb {R}}^2_+$,

$$\begin{aligned} A&:= \begin{pmatrix} 0 &{} \varepsilon ' (1-\mu )\\ 0 &{} (1-\varepsilon ') (1-\mu ) \end{pmatrix}, \quad b:= \begin{pmatrix} 1-\varepsilon \\ \varepsilon \end{pmatrix}, \quad c^*:= \begin{pmatrix} 1&0 \end{pmatrix}\,, \\ v&:= \begin{pmatrix} v_1 \\ v_2 \end{pmatrix}, \quad f(w,y) := g(wy) \,, \end{aligned}$$

so that assumptions (L1) and (L2) are satisfied. The matrix $A+bc^*$ is strictly positive (meaning every entry is positive) and consequently (P3) holds, whence (P1) does as well by Lemma 4.5. It is straightforward to compute that

6.2 $$\begin{aligned} {{\mathbf {G}}}(1)=\frac{\mu (1-\varepsilon ) +\varepsilon '(1-\mu )}{\mu +\varepsilon '(1-\mu )}=:\frac{1}{p}\,. \end{aligned}$$

In the following we consider Beverton–Holt and Ricker nonlinearities in (6.1) separately.

*Case I: Beverton–Holt* Set $g(y)=\lambda y/(1+ky)$ for fixed $k>0$. Using Proposition 4.3, we see that *f* always satisfies condition (N1), and condition (N2) holds if $\lambda u^- > 1/{{\mathbf {G}}}(1)=p$. Additionally, fixing $u^{\mathrm{e}} = 1$, condition (N3) holds if $\lambda u^- > 1/{{\mathbf {G}}}(1)=p$. Indeed, Franco et al. (2017), Table 5.1 shows that (5.1) is satisfied and a straightforward calculation yields

$$\begin{aligned} 0<\frac{\partial f}{\partial y}(1,y^{\mathrm{e}})=\frac{f(1,y^{\mathrm{e}})}{y^{\mathrm{e}}(1+ky^{\mathrm{e}})}= \frac{p}{1+ky^{\mathrm{e}}}<p,\quad \text{ where }\,\,\, y^{\mathrm{e}}=\frac{\lambda -p}{kp}, \end{aligned}$$

implying that (5.2) holds.

Thus, we can use Theorem 4.4 and Corollary 5.3 to obtain boundedness, persistence and stability results for (6.1). In order to compare our results with those in Chow et al. (2018), assume that $\mu =0$. It follows from (6.2) that the condition ${{\mathbf {G}}}(1)=1$ and $\lambda >1$ is sufficient for the existence of a unique positive equilibrium $x^{\mathrm{e}}$ of (6.1) with $u = 1$ and $v_1 = v_2 = 0$, and $x^{\mathrm{e}}$ is stable and attracts every solution with a nonnegative and nonzero initial condition. Indeed, for each compact subset $\Gamma \subseteq {\mathbb {R}}^2_+$ which does not contain zero, the rate of convergence of *x*(*t*) to $x^{\mathrm{e}}$ is exponential for all $x^0 \in \Gamma$, with constants depending on $\Gamma$ and the model data, but not on $x^0$. These conclusions extend (Chow et al. (2018), Theorem 3.1 b), where only symmetric dispersal ($\varepsilon '=\varepsilon$) was considered in the Beverton–Holt case in the absence of forcing. Corollary 5.3 indicates that dispersal, captured by $\varepsilon$ and $\varepsilon '$ (symmetric or not) has no effect on the global stability of the population when there is no attrition in the refuge, (that is, when $\mu = 0$). This property was noted in Chow et al. (2018) for the particular case of symmetric dispersal.

If there is attrition in the refuge, (that is $\mu \ne 0$) then the condition

$$\begin{aligned} \lambda >p= \frac{\mu +\varepsilon '(1-\mu )}{\mu (1-\varepsilon ) +\varepsilon '(1-\mu )}. \end{aligned}$$

guarantees that (N3) holds, and hence Corollary 5.3 is applicable. In particular, for a fixed value $\mu \in (0,1)$, dispersal has a direct effect on the range of growth-rate parameters $\lambda$ guaranteeing the convergence of all non-zero initial conditions to a positive equilibrium. Observe that $p = p(\varepsilon ,\varepsilon ')$ is an increasing function in $\varepsilon$ (dispersal into refuge), and a decreasing function in $\varepsilon '$ (dispersal from refuge). Consequently, when there is mortality in the refuge it is advantageous, from a perspective of ensuring population persistence, if there is less dispersal into the refuge, and more dispersal out of the refuge.

*Case II: Ricker* We now consider (6.1) with the Ricker-type function $g(y)=\lambda ye^{-y}$, and seek to compare our results with the numerical findings in Newman et al. (2002). There are no analytical results for this choice of nonlinear term in Newman et al. (2002). Let $u^{\mathrm{e}}\in [u^-,u^+]$ and consider the inequalities

6.3 $$\begin{aligned} \lambda u^+ e^{-2}< p < \lambda u^-\,. \end{aligned}$$

In Newman et al. (2002) it is assumed that

6.4 $$\begin{aligned} \mu = 0, \quad \varepsilon =\varepsilon ', \quad u^- = u^+ = u^{\mathrm{e}} = 1, \quad v_1= v_2 = 0\,. \end{aligned}$$

First note that, with model parameters as in (6.4), it follows from (6.2) that $p=1$, and so by Theorem 2.3, we have that *x*(*t*) converges to zero (exponentially) when $\lambda <1$.

Next, if the second inequality in (6.3) is satisfied, which simplifies to $1 <\lambda$ under the model assumptions (6.4), then condition (N2) holds and Theorem 4.4 ensures boundedness and persistence of (6.1). The persistence result validates the observation in Newman et al. (2002), p. 126 that: “[for large $\lambda$] the system is inherently stable for all values of the initial densities, in that the population number is always finite and positive.”

To consider stability of (6.1), now assume that (6.3) holds. Then the unique positive solution $y^{\mathrm{e}}$ of $f(u^{\mathrm{e}}, y) = g(u^{\mathrm{e}} y) = py$ is given by

$$\begin{aligned} y^{\mathrm{e}} = \frac{\ln (\lambda u^{\mathrm{e}}/p)}{u^{\mathrm{e}}}>0, \end{aligned}$$

and it is not difficult to see that (5.1) is satisfied (cf. Franco et al. (2017), Table 5.1). Furthermore, a routine calculation gives

$$\begin{aligned} \frac{\partial f}{\partial y}(u^{\mathrm{e}},y^{\mathrm{e}})=p(1-u^{\mathrm{e}}y^{\mathrm{e}})=p\big (1-\ln (\lambda u^{\mathrm{e}}/p)\big ), \end{aligned}$$

and it follows from (6.3) that $|(\partial f/\partial y)(u^{\mathrm{e}},y^{\mathrm{e}})|<p$. Consequently, (5.2) holds and so we have established that, under the assumption (6.3), condition (N3) is satisfied. Thus, we can invoke Corollary 5.3 to conclude stability of (6.1) provided that (6.3) holds. These stability conclusions supports the numerical simulations presented in Newman et al. (2002), Figure 5, where it is observed that the population dynamics tend to a constant positive population size for *small* values of $\lambda >1$ (recall that we have already seen that $\lambda >1$ is necessary for persistence).

We conclude the example with an illustrative numerical simulation. For the choice of parameters

6.5 $$\begin{aligned} \lambda =6, \quad \varepsilon =0.2, \quad \varepsilon '=0.3, \quad \mu = 0, \quad u^-=0.9, \quad u^{\mathrm{e}} =1, \quad u^+=1.1\,, \end{aligned}$$

the condition (6.3) holds and so Corollary 5.3 is applicable.

By Lemma 5.1, the unique positive equilibrium of (6.1) (for $u = u^{\mathrm{e}}$ and $v = 0$) is given by

6.6 $$\begin{aligned} x^{\mathrm{e}} := (I-A)^{-1}b p y^{\mathrm{e}} = \frac{\ln (\lambda u^{\mathrm{e}}/p)}{u^{\mathrm{e}}}\begin{pmatrix} 1 \\ \varepsilon \big (\mu (1-\varepsilon )+\varepsilon '(1 - \mu )\big )^{-1} \end{pmatrix}. \end{aligned}$$

**Fig. 2:**
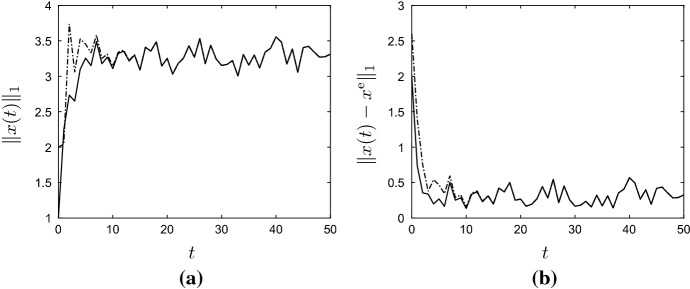
Graphs of state $\Vert x(t)\Vert _1$ (**a**) and state error $\Vert x(t) - x^{\mathrm{e}}\Vert _1$ (**b**) against *t* for the active and refuge population model (6.1) from Example 6.1. The parameter values and initial conditions are as in (6.5) and (6.7), respectively, and $g(y)=\lambda y e^{-y}$. The solid and dashed lines correspond to the first and second initial condition in (6.7), respectively

Figure 2 contains plots of $\Vert x(t) \Vert _1$ and $\Vert x(t)-x^{\mathrm{e}}\Vert _1$ against *t* for randomly determined $u(t) \in [u^-, u^+]$ and $v_1(t),v_2(t) \in [0,0.2]$, and for the two initial conditions

6.7 $$\begin{aligned} x^0 =\begin{pmatrix} 1\\ 0 \end{pmatrix} \quad \text {and} \quad x^0 = \begin{pmatrix} 0\\ 2 \end{pmatrix}\,. \end{aligned}$$

**Fig. 3:**
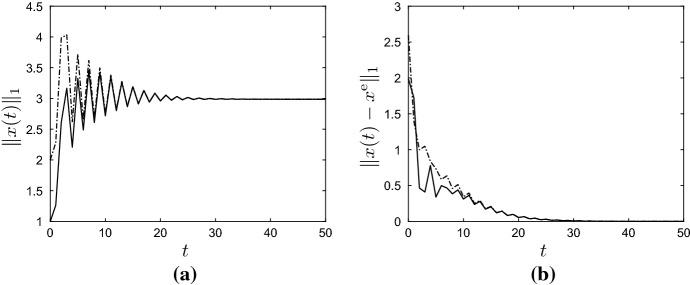
Graphs of state $\Vert x(t)\Vert _1$ (**a**) and state error $\Vert x(t) - x^{\mathrm{e}}\Vert _1$ (**b**) against *t* for the active and refuge population model (6.1) from Example 6.1. The parameter values are as in (6.5) and $g(y)=\lambda y e^{-y}$. Here *u* and *v* are as in (6.8), and the solid and dashed lines correspond to the first and second initial condition in (6.7), respectively

In particular, Fig. 2 shows that the impact of the initial vector $x^0$ on the evolution of *x*(*t*) is disappearing for large *t*, as was to be expected by Corollary 5.3. To illustrate the convergence result, statement (b) of Theorem 5.2, Fig. 3 contains plots of $\Vert x(t) \Vert _1$ and $\Vert x(t)-x^{\mathrm{e}}\Vert _1$ against *t* for *u* and *v* given by

6.8 $$\begin{aligned} \left. \begin{aligned} u(t)&= u^\mathrm{e} + (-0.8)^t=1+(-0.8)^t, \\ v(t)&= (0.7)^t\begin{pmatrix} 0.2 \\ 0.2 \end{pmatrix}, \end{aligned}\right\} \quad \forall \, t \in {\mathbb {Z}}_+\,, \end{aligned}$$

and for the two initial conditions in (6.7). Clearly, $u(t) \rightarrow 1$ and $v(t) \rightarrow 0$ as $t \rightarrow \infty$, and the figures show that $x(t)\rightarrow x^{\mathrm{e}}$ as $t\rightarrow \infty$. Note that the exact functional form of *u* and *v* in (6.8) is unimportant — it is their limiting properties which are crucial. $\Diamond$

#### Example 6.2

We consider a population structured in three groups, namely, juveniles, members of the population who are in a dormant state, which we shall refer to as “dormants”, and adults. These stage-classes are denoted by $x_1$, $x_2$ and $x_3$, respectively. We assume that after one time-step the population of juveniles splits between dormants and adults at a fixed rate $\alpha$. Dormants remain inactive for one time-step and then they become adults with a fixed rate $\beta$ or die. Juveniles are produced by adults following a density-dependent reproduction given by a nonlinear function *g*.

**Fig. 4:**
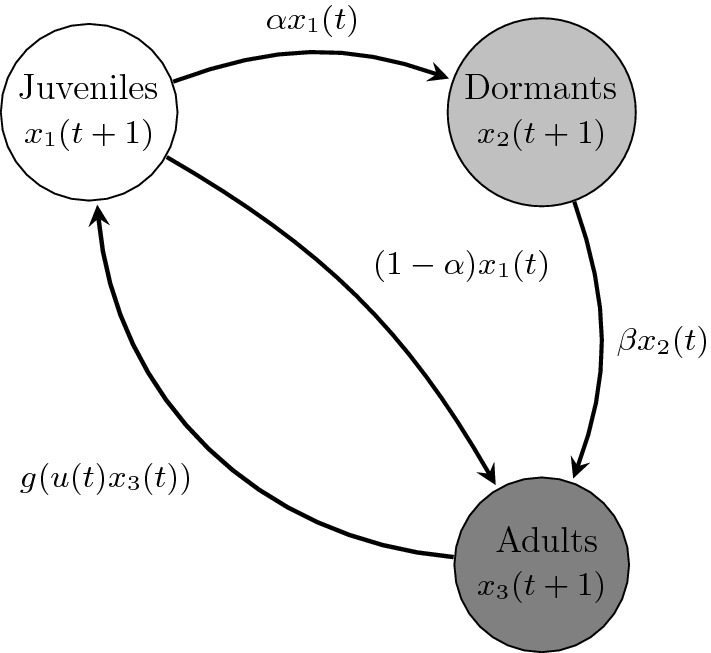
Schematic illustration of the population model (6.9)

A schematic representation of the dynamics of the population appears in Fig. 4. The following system models such a population,

6.9 $$\begin{aligned} \left. \begin{aligned} x_1(t+1)&= g(u(t) x_3(t))&x_1(0)&= x_1^0,\\ x_2(t+1)&= \alpha x_1(t)&x_2(0)&= x_2^0,\\ x_3(t+1)&= (1-\alpha ) x_1(t) + \beta x_2(t)&x_3(0)&= x_3^0, \end{aligned} \right\} \quad \forall \,t \in {\mathbb {Z}}_+\,, \end{aligned}$$

where $\alpha ,\beta \in (0,1)$ and, as before, the forcing $u(t) \in U:=[u^-,u^+]\subset (0,\infty )$ models the effect of demographic fluctuations affecting recruitment. The inclusion of dormancy in a population model is natural for several reasons. First, a large number of plants from a wide range of habitats are known to have persistent seed banks, that is, seeds can remain dormant for more than a generation (Thompson and Grime 1979). Second, dormancy is known to affect the stability of the population (MacDonald and Watkinson 1981). Finally, a large fraction of the total population may be present as dormant individuals and therefore ignoring it could give a poor estimate of the size of the population (Begon et al. 2009).

System (6.9) can be rewritten in the form (1.1) with

$$\begin{aligned} A=\left( \begin{matrix} 0 &{} 0 &{} 0\\ \alpha &{} 0 &{} 0\\ 1-\alpha &{} \beta &{} 0 \end{matrix} \right) , \quad b=\left( \begin{matrix} 1\\ 0\\ 0 \end{matrix} \right) , \quad c^*=\left( \begin{matrix} 0&0&1 \end{matrix} \right) \quad \text{ and } \,\,f(w,y)=g(wy). \end{aligned}$$

Clearly, conditions (L1) and (L2) hold. Moreover, condition (P1) holds with $\tau =3$. Verifying the nonlinear assumptions (N1)–(N3) of course requires some properties of *g*, but should they be satisfied then one can use Theorem 4.4 and Corollary 5.3 to study boundedness, persistence and stability properties of model (6.9). Note that $c^* b=0$ and for any

$$\begin{aligned} z=\begin{pmatrix} 0\\ 0\\ z_0^3 \end{pmatrix}\,, \end{aligned}$$

with $z_0^3\ge 0$ we have $c^* Az=0$. Therefore, the assumptions on *A*, *b* and $c^*$ required in Smith and Thieme (2013), Theorems 4.4, 5.8 (b) do not hold, and hence, even in the absence of forcing, these results are not applicable to (6.9). $\Diamond$

### Infinite-dimensional examples

We conclude the paper with two infinite-dimensional examples to which we apply the theory developed in Sects. 4 and 5. The first example considers the class of IPM models and the second provides a numerical illustration of the abstract theory in the context of an IPM coupled to a two-dimensional difference equation.

#### Example 6.3

An exemplar for the infinite-dimensional theory developed in this paper is a class of integro-difference equations, the so-called integral projection models. IPMs were introduced as a tool for ecological modelling in Easterling et al. (2000), see also Childs et al. (2003), Ellner and Rees (2006), and the reader is referred to Merow et al. (2014) for a recent review. They are relevant when the population stages are naturally described by a continuous variable, such as size or weight, as opposed to discrete stage-classes, such as insect instars. The purpose of the present example is to interpret the assumptions (L1), (L2), (P1) and (N1) –(N3) in the context of IPMs.

A forced linear IPM typically takes the form

6.10 $$\begin{aligned} \left. \begin{aligned} n(t+1,\xi )&= \int _\Omega k(\xi , \zeta ) n(t, \zeta ) \, d \zeta + m(t,\xi ) \quad \forall \, t \in {\mathbb {Z}}_+, \\ n(0, \xi )&= n^0(\xi ), \end{aligned}\right\} \quad \text {almost all} \; \xi \in \Omega \,, \end{aligned}$$

where $\Omega$ is a measurable subset of ${\mathbb {R}}^q$ and $n(t, \cdot )$ denotes the population distribution at time-step *t*, with initial distribution $n^0$. Further, *m* denotes an additive forcing term which models, say, the effects of migration, breeding or planting schemes. The kernel *k* captures survival, growth, and recruitment of the population. We refer the reader to Merow et al. (2014) for more information on how kernels are constructed and parametrised in practice. In particular, following Rebarber et al. (2012), the kernel *k* is often assumed to be of the form $k = \rho + \sigma$, where $\rho$ and $\sigma$ denote a survival and recruitment term, respectively. Specifically, $\rho (\xi ,\zeta )$ denotes the probability that an individual at stage $\zeta$ survives or grows to an individual at stage $\xi$ in one time-step, and so is evidently nonnegative-valued. In applications $\rho$ is typically continuous and $\Omega$ is compact. We shall assume that $\rho \in L^\infty (\Omega \times \Omega , {\mathbb {R}}_+)$. In the presence of mortality in the population, the assumption

6.11 $$\begin{aligned} {{\,\mathrm{ess\,sup}\,}}_{\zeta \in \Omega } \int _{\Omega } \rho (\xi ,\zeta ) \, d \xi <1\,, \end{aligned}$$

is usually satisfied.

To write (6.10) as an infinite-dimensional difference equation, the natural choices for the state space *X* and the cone *C* are $X := L^1(\Omega , {\mathbb {R}})$ and $C := L^1(\Omega , {\mathbb {R}}_+)$. Defining the integral operator $A : X \rightarrow X$ by

6.12 $$\begin{aligned} (A z)(\cdot ) := \int _{\Omega } \rho (\cdot \,, \zeta )z(\zeta ) d \zeta \quad \forall \, z\in X, \end{aligned}$$

it follows from (6.11) that $A\in {\mathcal {L}}(X)$ and $\Vert A\Vert <1$ (Lax 2002, Theorem 1, Chapter 16), and so *A* is exponentially stable. It is clear that *A* leaves *C* invariant, that is, *A* is positive. We have now established that *A* satisfies (L1). Assuming that the recruitment term $\sigma$ can be expressed as

$$\begin{aligned} \sigma (\xi ,\zeta ) = b(\xi ) \nu c(\zeta ), \quad \text {almost all} \; (\xi ,\zeta ) \in \Omega \times \Omega \,, \end{aligned}$$

where $b \in C$ denotes the distribution of new individuals, $\nu >0$ is an offspring survival probability over one time-step, and $c(\zeta )$ denotes the per-capita fecundity of an individual at stage $\zeta$, the distribution of offspring in one-step from distribution $z\in X$ is given by

6.13 $$\begin{aligned} \int _{\Omega } \sigma (\cdot \,,\zeta ) z(\zeta ) \, d\zeta = b(\cdot ) \nu \int _{\Omega } c(\zeta ) z(\zeta )\,d\zeta =b(\cdot )\nu c^*z\,. \end{aligned}$$

Note that

6.14 $$\begin{aligned} c^* z := \int _{\Omega } c(\zeta ) z(\zeta )\,d \zeta \end{aligned}$$

is the total number of offspring produced in a single time-step by the distribution $z\in X$. Imposing the natural assumption that $c \in L^\infty (\Omega , {\mathbb {R}}_+)$, it follows that $c^* \in C^*$. Setting $x(t) : = n(t,\cdot \,)$, in light of (6.12)–(6.14), it follows that (6.10) may be expressed as

6.15 $$\begin{aligned} x^\nabla = (A+b \nu c^*)x + v, \quad x(0) = n^0\,, \end{aligned}$$

where $v(t)=m(t,\cdot \,)$ and we assume that $m(t,\cdot \,)\in C$ for every $t\in {\mathbb {Z}}_+$. Since $b \in C$ and $c^* \in C^*$, we have that (L2) is satisfied (provided that $b\ne 0$ and $c^* \ne 0$).

A nonlinear term arises in (6.15) if the survival probability $\nu$ depends on the total number of offspring produced in a single time-step, see (6.14). In this case, the linear operator induced by the kernel $\sigma$ is replaced by the nonlinear operator

$$\begin{aligned} z\mapsto bg\left( \int _{\Omega } c(\theta ) z(\theta )\,d \theta \right) \int _\Omega c(\zeta )z(\zeta )\,d \zeta =bg(c^*z)c^*z \,, \end{aligned}$$

where *b*, *c* and $c^*$ are as before and *g* denotes the probability of survival of new individuals over one time-step. The function *g* models density-dependence in recruitment, reflecting competition effects at higher abundance, for instance. Consequently, we obtain

6.16 $$\begin{aligned} x^\nabla = Ax+bg(c^*x)c^*x + v = A x + b f(c^* x) +v, \quad x(0) = n^0\,, \end{aligned}$$

where $f(y) = g(y)y$ and *v* is as before. Evidently, both (6.15) and (6.16) are special cases of (1.1).

The nonlinear assumptions (N1)–(N3) depend on the interplay between *f* in (6.16) and the constant $p = 1/{{\mathbf {G}}}(1)$. We proceed to discuss the key additional assumption (P1) required for the persistence and stability results in Sects. 4 and 5 . By part (b) of Example 2.2, (P1) holds with $\tau =0$ if, and only if ${{\,\mathrm{ess\,inf}\,}}_{\theta \in \Omega } c(\theta ) >0$. To discuss the case wherein the integer $\tau$ is positive, let *k* denote the kernel of $A + bc^*$, that is,

$$\begin{aligned} k(\xi ,\zeta ) = \rho (\xi , \zeta ) + b(\xi )c(\zeta )\,, \end{aligned}$$

and, for $n\in {\mathbb {N}}$, define $k_n$ recursively via $k_1:=k$ and

$$\begin{aligned} k_{n+1}(\xi ,\zeta ) = \int _\Omega k(\xi , s) k_n(s,\zeta )\,ds \quad \forall \,n \in {\mathbb {N}}. \end{aligned}$$

It is clear that (P1) holds for a non-zero integer $\tau$ if, and only if,

6.17 $$\begin{aligned} {{\,\mathrm{ess\,inf}\,}}_{\zeta \in \Omega } \int _{\Omega } c(s) k_\tau (s , \zeta ) \, ds >0\,. \end{aligned}$$

If *c* and *k* are continuous, then (6.17) holds if $\mathrm{supp}\, c \, \cap \, \mathrm{supp}\, k_\tau (\cdot ,\zeta ) \ne \emptyset$ for all $\zeta \in \Omega$ or, equivalently, for every $\zeta \in \Omega$, there exists $s \in \Omega$ such that $c(s) k_\tau (s,\zeta ) >0$.

To the best of our knowledge, the paper Rebarber et al. (2012) was the first to consider unforced IPMs and model them in the abstract infinite-dimensional state-space form (1.1) (with $f(u,y)=f(y)$ and $v=0$), and has in part inspired the present work. The notions of boundedness and persistence do not play a prominent role in Rebarber et al. (2012) and, as mentioned, Rebarber et al. (2012) does not consider forced systems. Unfortunately, the IPM result (Rebarber et al. 2012, Corollary 4.1) is not correct, see Appendix B. $\Diamond$

#### Example 6.4

We consider a model for the Clonal Perennial Herb (*Veratrum album*) from Hesse et al. (2008). The model contains an IPM coupled to two-dimensional difference equations, namely

6.18 $$\begin{aligned} \left. \begin{aligned} n(t+1,\xi )&= D(t) p_{\mathrm{sc}} f_{\mathrm{sd}}(\xi ) + \int _{m_1}^{m_2} k(\xi , \zeta ) n(t, \zeta ) \, d \zeta \\ D(t+1)&= p_{\mathrm{est}}\big ( g_0 h^*n(t, \cdot ) + g_1 S_1(t)\big ) \\ S_1(t+1)&= (1-g_0)s_0 h^*n(t, \cdot ) \end{aligned} \right\} \quad \forall \, t \in {\mathbb {Z}}_+, \; \forall \, \xi \in [m_1, m_2]\,. \end{aligned}$$

The variable $n(t, \xi )$ is the number of plants with (natural logarithm of) shoot diameter equal to $\xi$ (in mm) at discrete time-step $t \in {\mathbb {Z}}_+$. Following Hesse et al. (2008), we assume that the variable $\xi$ takes minimum and maximum values given by $m_1 = 0$ and $m_2 = 3.5$ (and so $e^{m_1} = 1$mm and $e^{m_2} = 33$mm), respectively, and that the time-steps correspond to years. We set $\Omega := [m_1,m_2]$ and, consequently, $n(t, \cdot ) \in X_0:= L^1(\Omega , {\mathbb {R}})$ for each $t \in {\mathbb {Z}}_+$. The variables *D* and $S_1$ in (6.18) denote the abundance of the seedling/cotyledon stage-class of *Veratrum*, and the number of 1-year old seeds, respectively.^3^ The terms $g_0$ and $g_1$ are probabilities of new and 1-year old seed germination, respectively, whilst $s_0$ is the probability of new seed survival. The term $p_{\mathrm{sc}}$ is the probability of an individual cotyledon growing to the juvenile stage-class in one time-step, and those that do are distributed according to $f_{\mathrm{sd}} \in C_0$, where $C_0\subset X_0$ is the cone $C_0=L^1(\Omega ,{\mathbb {R}}_+)$. Moreover, $p_{\mathrm{est}}$ denotes the probability of a seed establishing as a cotyledon.

The kernel *k* of the integral operator in (6.18) is given by

6.19 $$\begin{aligned} k(\xi , \zeta ) = \rho (\xi , \zeta ) + p_{\mathrm{s}}(\zeta ) p_{\mathrm{f}}(\zeta ) f_{\mathrm{v}}(\zeta ) f_{\mathrm{vd}}( \xi , \zeta ) \quad \forall \, \xi , \zeta \in \Omega \,, \end{aligned}$$

where $\rho$ is the probability of an individual of size $\zeta$ surviving to one of size $\xi$ in a single time-step. It is assumed that $\rho$ has the form

6.20 $$\begin{aligned} \rho (\xi ,\zeta ) = p_{\mathrm{s}}(\zeta )(1-p_{\mathrm{f}}(\zeta ))g(\xi ,\zeta ) \quad \forall \,\xi , \zeta \in \Omega \,, \end{aligned}$$

where $p_{\mathrm{s}}(\zeta )$ and $p_{\mathrm{f}}(\zeta )$ are the survival probability and flowering probability of an individual of size $\zeta$, respectively, and $g(\xi ,\zeta )$ is the probability of an individual of size $\zeta$ growing to size $\xi$, each over one time-step. The term $1- p_{\mathrm{f}}$ appears on the right-hand side of (6.20) as flowering is fatal to *Veratrum*, that is, it is monocarpic.

The second summand on the right-hand side of (6.19) captures asexual reproduction of *Veratrum*: $f_{\mathrm{v}}(\zeta )$ denotes the number of asexual offspring produced by an individual of size $\zeta$, where $f_{\mathrm{vd}}(\xi , \zeta )$ is the probability that an individual of size $\zeta$ asexually produces an offspring of size $\xi$ in one time-step.

The functional $h^*$ in (6.18) is given by

$$\begin{aligned} h^*z= \int _{\Omega }h(\zeta )z(\zeta )\, d \zeta \,\,\,\,\forall \,z\in X_0,\quad \text{ where } \,\,h(\zeta ):= p_{\mathrm{f}}(\zeta ) f_{\mathrm{s}}(\zeta ) p_{\mathrm{s}}(\zeta ). \end{aligned}$$

Here $f_{\mathrm{s}}(\zeta )$ denotes the expected number of seeds produced by an individual of size $\zeta$. Note that $h^*z$ denotes the total number of seeds produced by the distribution *z* in a single time-step.

The functional forms for $p_{\mathrm{s}}$, $p_{\mathrm{f}}$, *g*, $f_{\mathrm{v}}$, and $f_{\mathrm{vd}}$ are as in Hesse et al. (2008), Table 1. We use the parameter values

$$\begin{aligned} a_{\mathrm{s}}&= -4.53,&b_{\mathrm{s}}&= 5.51,&c_{\mathrm{s}}&= 1.04,&a_{\mathrm{g}}&= 0.28,&b_{\mathrm{g}}&= 0.92, \\ \beta _{\mathrm{g}}&= 0.44,&\beta _0&= -7.08,&\beta _{\mathrm{s}}&= 1.5,&a_{\mathrm{v}}&= -3.2,&b_{\mathrm{v}}&= 1.24, \\ a_{\mathrm{vd}}&= 1.16,&b_{\mathrm{vd}}&= 0.48,&\sigma ^2_{\mathrm{vd}}&= 0.18,&g_0&= 0.19,&g_1&= 0.98,\\ s_0&= 0.91,&s_1&= 0.98,&p_{\mathrm{est}}&= 0.31. \end{aligned}$$

which are equal to, or within one standard error of, the statistical estimates in Hesse et al. (2008), Table 1, Pasture environment. Further, $f_{\mathrm{s}}(s) = \mathrm{e}^{1.5 + 1.4 s}$ (cf. Hesse et al. (2008), p. 202, column 2) and $f_{\mathrm{sd}}$ is assumed normally distributed with mean 1.05 and variance 0.72 (cf. Hesse et al. (2008), p. 203, column 1).^4^ In particular, with these choices, the function *h* is in $L^\infty (\Omega , {\mathbb {R}}_+)$, and so $h^*\in C_0^*$.

The results of the current paper apply to the model (6.18). Since (6.18) is both linear and unforced, however, we proceed to demonstrate how the present results apply to a variation of the above model which includes these extra features. First, we assume that the cotyledon stage-class *D*(*t*) is subject to exogenous forcing $\nu (t)\ge 0$, which could be the result of a planting scheme, or dispersal. Second, seeking to capture density-dependent crowding effects which occur at higher abundances, we assume that the constant probability $p_{\mathrm{sc}}$ which appears in (6.18) is in fact a non-increasing function of *D*, and that this probability is subject to some seasonal or anthropogenic variation $u(t)>0$ with nominal value $u^{\mathrm{e}} =1$. Modifying (6.18) in this way, leads to the following system.

6.21 $$\begin{aligned} \left. \begin{aligned} n(t+1,\xi )&= D(t)p_{\mathrm{sc}}(u(t)D(t))f_{\mathrm{sd}}(\xi ) + \int _{m_1}^{m_2} k(\xi , \zeta ) n(t, \zeta ) \, d \zeta \\ D(t+1)&= p_{\mathrm{est}}\big ( g_0 h^*n(t, \cdot ) + g_1 S_1(t)\big )+\nu (t) \\ S_1(t+1)&= (1-g_0)s_0 h^*n(t, \cdot ) \end{aligned} \right\} \quad \forall \, t \in {\mathbb {Z}}_+, \; \forall \, \xi \in [m_1, m_2]\,. \end{aligned}$$

To write (6.21) in the form (1.1), it is natural to introduce the state space $X := X_0\times {\mathbb {R}}^2$ which is a Banach space when equipped with the norm

$$\begin{aligned} \left\| \begin{pmatrix} z_0 \\ z_1 \end{pmatrix} \right\| _X := \Vert z_0\Vert _{X_0} + \Vert z_1 \Vert _{1} \quad \forall \,(z_0,z_1)\in X. \end{aligned}$$

The dual space of *X* can be identified with $X^* = X_0^* \times {\mathbb {R}}^2$. In the given setting, the natural cone $C\subset X$ to consider is $C:=C_0 \times {\mathbb {R}}^2_+$. This cone is reproducing and its dual cone $C^*$ can be identified with $C_0^* \times {\mathbb {R}}^2_+$ and it is clear that the interior of $C^*$ is non-empty, see Example 2.2.

Defining the integral operator $A_0 : X_0\rightarrow X_0$ by

$$\begin{aligned} (A_0z)(\xi ) = \int _{\Omega } k (\xi , \zeta )z(\zeta ) d \zeta \quad \forall \,z \in X_0\,, \end{aligned}$$

the assumptions on *k* ensure that *k* is continuous and non-negative, and therefore $A_0$ is a bounded positive operator (Lax 2002, Theorem 1, Chapter 16). We proceed to introduce the linear system $(A,b,c^*)$ which underlies the abstract formulation of (6.18). To this end, we define a positive bounded linear operator $A : X \rightarrow X$ by

6.22 $$\begin{aligned} Az := \begin{pmatrix} A_0 &{} 0 &{} 0 \\ p_{\mathrm{est}}g_0 h^* &{} 0 &{} p_{\mathrm{est}} g_1 \\ (1-g_0)s_0 h^* &{} 0 &{} 0 \end{pmatrix} z, \quad \forall \, z \in X\,, \end{aligned}$$

and set

6.23 $$\begin{aligned} b := \begin{pmatrix} f_{\mathrm{sd}} \\ 0 \\ 0 \end{pmatrix},\quad c^*z:= \begin{pmatrix} 0&1&0 \end{pmatrix}z,\,\,\, \forall \,z\in C. \end{aligned}$$

It is clear that $b\in C$ and $c^* \in C^*$, and so (L2) is satisfied. The forced nonlinear system (6.18) may be written as

6.24 $$\begin{aligned} x^\nabla =Ax+bp_{\mathrm{sc}}(uc^*x)c^*x+v,\quad x(0)=x^0\in C\,, \end{aligned}$$

where

$$\begin{aligned} x(t) : = \begin{pmatrix} n(t, \cdot ) \\ D(t) \\ S(t) \end{pmatrix},\quad v(t):= \begin{pmatrix} 0 \\ \nu (t) \\ 0 \end{pmatrix}, \quad \forall \,t \in {\mathbb {Z}}_+\,. \end{aligned}$$

Clearly, (6.24) is a special case of (1.1) with $f : U\times {\mathbb {R}}_+ \rightarrow {\mathbb {R}}_+$ given by $f(u,y) := p_{\mathrm{sc}}(uy)y$.

From the block structure of *A*, we have that the spectral radii *r*(*A*) of *A* and $r(A_0)$ of $A_0$ coincide. With the given parameter values, we compute numerically that $r(A)=r(A_0) \approx 0.9413$, and so *A* is exponentially stable and (L1) holds. Ecologically, this means that asexual reproduction alone is not sufficient to maintain the population of *Veratum* asymptotically. For asymptotic population stasis or growth, a contribution from seed production and germination is required.

To show that assumption (P1) is satisfied, we compute that

$$\begin{aligned}&c^* (A + bc^*)^3 \\&\quad = p_{\mathrm{est}}\,\mathrm{row}\begin{pmatrix} h^*\big ( g_0 A_0^2 + g_0^2 p_{\mathrm{est}} h^*(f_{\mathrm{sd}})I + g_1 (1-g_0)s_0 A_0 \big ) \\ h^* \big ( (g_0 A_0 + g_1(1- g_0)s_0 I )f_{\mathrm{sd}}\big ) \\ p_{\mathrm{est}} g_0 g_1 h^*(f_{\mathrm{sd}}) \end{pmatrix}, \end{aligned}$$

We note that (P1) cannot hold for $\tau <3$, as there exist $z_\tau \in C$ with $z_\tau \not =0$, such that $c^*(A+bc^*)^\tau z_\tau = 0$ for $\tau \in \{0,1,2\}$. By the choice of $p_{\mathrm{f}}$, $p_{\mathrm{s}}$ and $f_{\mathrm{s}}$, we have that ${{\,\mathrm{ess\,inf}\,}}h >0$, and thus, $h^* \in \mathrm{int}\,C_0 ^*$. Hence,

$$\begin{aligned} c^* (A + bc^*)^3 \ge p_{\mathrm{est}}\begin{pmatrix} g_0^2 p_{\mathrm{est}} h^*(f_{\mathrm{sd}}) h^*&g_1(1- g_0)s_0 h^*(f_{\mathrm{sd}})&p_{\mathrm{est}} g_0 g_1 h^*(f_{\mathrm{sd}}) \end{pmatrix} \in \mathrm{int}\, C^*, \end{aligned}$$

and so, by Lemma 2.1, $c^* (A + bc^*)^3 \in \mathrm{int}\,C^*$.

For the purpose of numerical simulations, we approximate (6.24) by using a finite-element method, the details of which are given in Appendix C. We numerically compute that ${{\mathbf {G}}}(1) \approx 87.8$, and so $p \approx 0.0114$. We assume that

6.25 $$\begin{aligned} p_{\mathrm{sc}}(y) = \frac{ q_{\mathrm{sc}}}{1 + 3y} \quad \forall \, y \ge 0\,, \end{aligned}$$

where $q_{\mathrm{sc}} = 0.52$. Since $q_{\mathrm{sc}}>p$, the function $f:U\times {\mathbb {R}}_+\rightarrow {\mathbb {R}}_+$ given by $f(w,y) = p_{\mathrm{sc}}(w y)y$ satisfies (5.1), see Franco et al. (2017), Example 4.1, Table 5.1, where $U\subset (0,\infty )$ is an arbitrary compact set such that $1\in U$. Moreover, the unique positive solution $y^{\mathrm{e}}$ of $f(1,y) = py$ is given by $y^{\mathrm{e}} = (q_{\mathrm{sc}} - p)/(3p) \approx 3.52$. Finally,

$$\begin{aligned} \frac{\partial f}{\partial y}(1,y_{\mathrm{e}}) = \frac{p}{1 + 3y^{\mathrm{e}}} \in (0,p)\,, \end{aligned}$$

whence (5.2) holds, and we conclude that the condition (N3) is satisfied. Consequently, the hypotheses of Corollary 5.3 are satisfied. The simulations shown below illustrate the conclusions of Corollary 5.3 in the context of the system under consideration.

**Fig. 5:**
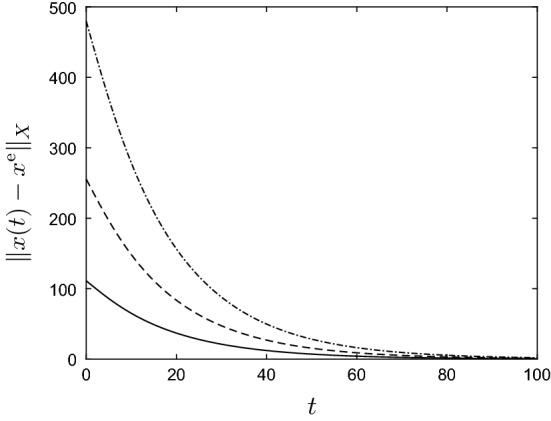
Graph of $\Vert x(t) - x^{\mathrm{e}}\Vert _X$ against *t* for the *Veratrum* model of Example 6.4. The simulations use three random initial conditions $x^0$ of increasing norm and forcing is absent ($u =1$, $v=0$)

**Fig. 6:**
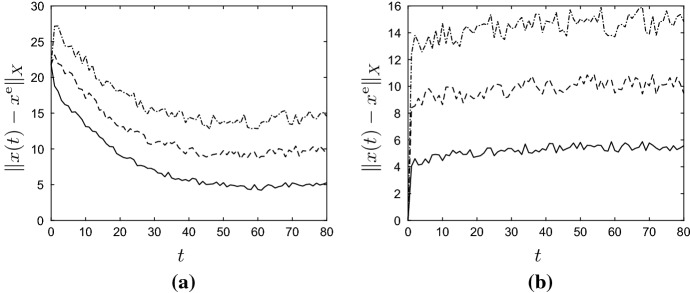
Graph of state errors against *t* for the *Veratrum* model of Example 6.4. The simulations in both panels use additive forcing $\nu _k$ from (6.26), with solid, dashed, and dashed-dotted lines corresponding to $k = 1,2,3$, respectively. Multiplicative forcing is absent ($u=1$) in these simulations and the initial conditions in panels (a) and (b) are 0 and $x^{\mathrm{e}}$, respectively

Figure 5 illustrates the semi-global exponential stability of $x^{\mathrm{e}}$—the state error $\Vert x(t) - x^{\mathrm{e}}\Vert _X$ is plotted against *t* for three randomly chosen non-zero initial conditions, in the absence of forcing forcing ($u = 1$, $v =0$). Figure 6a, b give an illustration of the ISS property of $x^{\mathrm{e}}$—it shows plots of the state errors against *t* for two different initial states (0 and $x^{\mathrm{e}})$, $u = 1$ and three different additive forcing terms $v_k$ with

6.26 $$\begin{aligned} \nu _k(t)= 4 k + \theta (t), \quad \forall \, t \in {\mathbb {Z}}_+, \quad k \in \{1,2,3\}\,, \end{aligned}$$

where $\theta$ is “small” random noise. Observe in Fig. 6a that the contribution to $\Vert x(t) - x^{\mathrm{e}}\Vert _X$ from the initial error $x(0) - x^{\mathrm{e}}=x^{\mathrm{e}}$ decays over time.

**Fig. 7:**
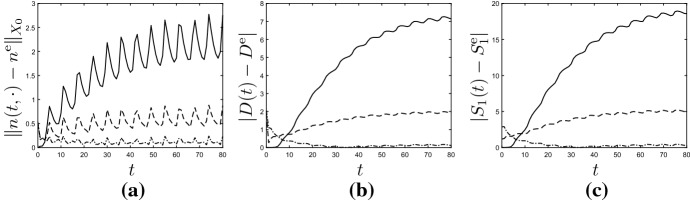
Graph of state errors against *t* for the *Veratrum* model of Example 6.4. The simulations use three initial conditions, zero additive forcing ($v =0$) and three periodic forcing $u_k$ given by (6.27), with solid, dashed, and dashed-dotted lines corresponding to $k = 1,2,3$, respectively. The solid line uses $x(0) = x^{\mathrm{e}}$ and the other two lines are randomly chosen non-zero initial conditions

Figure 7a–c show plots of the state errors against *t* for three different initial conditions, zero additive forcing ($v=0$), and three periodic multiplicative forcing terms $u_k$ given by

6.27 $$\begin{aligned} u_k(t) = \frac{ k}{2} (1 + 0.8 \sin t), \quad \forall \, t \in {\mathbb {Z}}_+, \quad k \in \{1,2,3\}\,. \end{aligned}$$

**Fig. 8:**
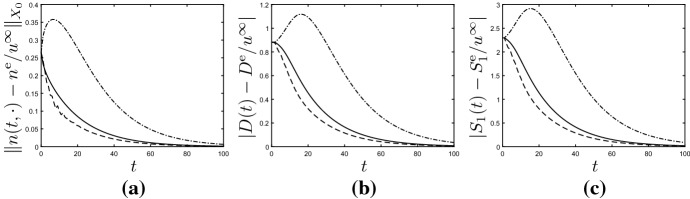
Graph of state errors against *t* for the *Veratrum* model of Example 6.4. The simulations all use initial condition $x^{\mathrm{e}}$, and three different pairs of forcing functions $(u_k(t),v_k(t))$ from (6.28), with solid, dashed, and dashed-dotted lines corresponding to $k = 1,2,3$, respectively

Whilst Figs. 5, 6, 7 illustrate different facets of our main stability result Theorem 5.2 and its Corollary 5.3, Fig. 8a–c provide an illustration of Corollary 5.6 by showing the convergence of *x*(*t*) to $x^{\mathrm{e}}/u^\infty$ for $u^\infty = 0.8$, when subject to three pairs of forcing functions $(u_k,v_k)$ given by

6.28 $$\begin{aligned} \left. \begin{aligned} u_1(t)&= u^{\infty }, \quad u_2 = u^{\infty }( 1 + (-0.8)^t \cos t), \quad u_3 = u^{\infty }( 1 + (0.9)^t), \\ v_k(t)&= k \theta (t) (0.9)^t, \end{aligned} \right\} \end{aligned}$$

where $k \in \{1,2,3\}$ and $\theta (t)$ is randomly drawn from [0, 1]. Evidently, for very $k\in \{1,2,3\}$, $u_k(t)$ and $v_k(t)$ converge to $u^\infty$ and 0, respectively, as $t \rightarrow \infty$.$\Diamond$

## Summary

We have presented boundedness, persistence and stability results for the class of nonlinear, forced difference equations (1.1) which arise frequently in population biology and theoretical ecology. Our treatment permits the situation wherein the state-space is infinite-dimensional, enabling the analysis of structured populations modelled with a finite number of stages as well as populations modelled with a continuum of stages (the latter are frequently described in terms of integro-difference equations, such as IPMs). The system (1.1) has both a linear and a nonlinear component, corresponding to a mixture of density-independent and density-dependent dynamical scenarios. A key feature of these models is that they are instances of positive systems, and so the state is constrained to lie in a positive cone. Under natural conditions these models admit two equilibria in the absence of forcing: zero and a non-zero steady state.

The current work continues a line of enquiry of the present authors, including Eager et al. (2014) and Franco et al. (2017), to develop a theoretical framework for the analysis of the effects of forcing in ecological modelling, analysis, and control. As discussed, the present work substantially extends Franco et al. (2017). We comment that the term forcing is used here to describe all exogenous signals, including disturbances and those affected by the user or modeller. Therefore, in a biological setting, the inclusion of forcing facilitates modelling a number of exogenous influences, including environmental or demographic variation, or management or intervention strategies, which are relevant in a number of application scenarios, from agriculture and conservation, to harvesting and pest management. We reiterate our motivation that forcing is typically *not* considered in the construction or analysis of nonlinear ecological models, yet forcing is arguably a persistent, poorly understood, and pernicious feature of these systems. Moreover, it is well-known that forcing can have hugely deleterious effects on the stability of nonlinear models, effects which are not well-predicted by stability analysis of the unforced model. Forcing or input functions play a key role in systems and control theory and, therefore, it is natural that our approach is inspired by the concept of input-to-state stability from nonlinear control theory.

Our main result for boundedness and persistence is Theorem 4.4, and our main stability results are Theorem 5.2, Corollaries 5.3 and 5.6. Informally, we establish our results by examining the interplay between the underlying linear system (2.4), particularly its transfer function “gain” ${{\mathbf {G}}}(1)=1/p$ (see Proposition 3.1), and the nonlinear term *f*. This interplay is captured in the nonlinear and sector-type assumptions (N1)–(N3), see Remark 4.2 as well. The persistence and stability results very much rely on the positivity property (P1) imposed on the linear system $(A,b,c^*)$ which ensures that the origin of (1.1) is repelling in a suitable sense. We comment that much attention in the ecology literature has been devoted to the importance of transient dynamics including, for example, Hastings (2004) and Stott et al. (2011). Our persistence results are uniform with respect to time, in contrast with other persistence concepts usually used in the literature [for example, Smith and Thieme (2011)], and therefore yield lower bounds for both transient and asymptotic dynamics.

In closing, we emphasise that our assumptions are not dependent on explicit knowledge of the model parameters, but rather impose (ecologically reasonable) structural requirements to be satisfied. For instance, there is considerable robustness in (N1)–(N3) with respect to the exact functional form of nonlinear term *f*, which is often difficult to parametrise. Section 6 contains detailed discussions of four non-trivial, biologically relevant examples of multi-stage, single species populations, in both finite- and infinite-dimensional settings. A natural future line of enquiry is study of multiple interacting populations, or communities, subject to external forcing.

## Proofs

In this appendix, we provide proofs of results stated in Sects. 2–5.

### Proofs for Sections 2 and 3

#### Proof of Lemma 2.1

- Assume that $x^*\in \mathrm{int}\,C^*$. Seeking a contradiction, suppose that (2.1) does not hold. Then there exist elements $\xi _n\in C$ with $\Vert \xi _n\Vert =1$, where $n\in {\mathbb {N}}$, and such that
  $$\begin{aligned} \varepsilon _n:=x^*(\xi _n)\rightarrow 0\quad \text{ as } n\rightarrow \infty \,. \end{aligned}$$
  Assume that there exists $k\in {\mathbb {N}}$ such that $\varepsilon _k=0$ and choose $y^*\in X^*$ such that $y^*(\xi _k)=1$ (existence of such a functional is guaranteed by the Hahn–Banach theorem). Then, for every $\delta >0$, the functional $z^*_\delta :=x^*-\delta y^*$ has the property $z^*_\delta (\xi _k)=-\delta <0$, and so $z^*_\delta \not \in C^*$, contradicting the hypothesis that $x^*\in \mathrm{int}\,C^*$. Consequently, $\varepsilon _n>0$ for all $n\in {\mathbb {N}}$. Invoking again the Hahn–Banach theorem, there exist $y_n^*\in X^*$ such that $\Vert y_n^*\Vert =1$ and $y^*_n(\xi _n)=1$ for all $n\in {\mathbb {N}}$. The functionals $z^*_n:=x^*-2\varepsilon _ny^*_n$ satisfy $z^*_n(\xi _n)=-\varepsilon _n<0$ and thus, $z^*_n\not \in C^*$. But $z^*_n\rightarrow x^*$ as $n\rightarrow \infty$, contradicting the hypothesis that $x^*\in \mathrm{int}\,C^*$.
  Conversely assume that (2.1) holds, in which case
  $$\begin{aligned} \varepsilon := \inf _{\xi \in C,\,\Vert \xi \Vert =1}x^*(\xi )>0\,. \end{aligned}$$
  Let $y^*\in X^*$ be such that $\Vert x^*-y^*\Vert <\varepsilon /2$. Then, for all $\xi \in C$ with $\Vert \xi \Vert =1$,
  $$\begin{aligned} y^*(\xi )=x^*(\xi )+(y^*-x^*)(\xi )\ge \varepsilon -\Vert y^*-x^*\Vert >\frac{\varepsilon }{2}\,. \end{aligned}$$
  Hence, $y^*(\xi )\ge (\varepsilon /2)\Vert \xi \Vert$ for all $\xi \in C$, showing that $y^*\in C^*$. Consequently, ${\mathbb {B}}(x^*,\varepsilon /2)\subset C^*$, establishing that $x^*\in \mathrm{int}\,C^*$.
- Statement (b) is an immediate consequence of statement (a). $\square$

#### Proof of Proposition 3.1

- It follows from (L1) and (L2) that
  $$\begin{aligned} {{\mathbf {G}}}(1)=c_{\mathrm{c}}^*(I-A_{\mathrm{c}})^{-1}b=c^*(I-A)^{-1}b=\sum _{k=0}^\infty c^*A^kb\ge 0\,. \end{aligned}$$
  Furthermore, a straightforward calculation yields that, for every $z\in {\mathbb {C}}$ with $|z|\ge 1$,
  $$\begin{aligned} |{{\mathbf {G}}}(z)|=\frac{1}{|z|}|c^*_{\mathrm{c}}(I-(1/z)A_{\mathrm{c}})^{-1}b|\le \sum _{k=0}^\infty \frac{1}{|z|^{k+1}}c^*A^kb \,. \end{aligned}$$
  Combining this with the identity
  $$\begin{aligned} |{{\mathbf {G}}}(1)|={{\mathbf {G}}}(1)=c^*(I-A)^{-1}b=\sum _{k=0}^\infty c^*A^kb\,, \end{aligned}$$
  shows that $$\Vert {{\mathbf {G}}}\Vert _{H^\infty }={{\mathbf {G}}}(1)$$.
- This follows from standard stability radius theory.
- A straightforward calculation shows that
  $$\begin{aligned} \big (A + pbc^*\big ) (I-A)^{-1} b = (I-A)^{-1} b\,. \end{aligned}$$
  Invoking (L1) and (L2), we see that
  $$\begin{aligned} (I-A)^{-1} b = \sum _{j=0}^\infty A^j b \ge b \ne 0\,, \end{aligned}$$
  whence 1 is an eigenvalue of $A_{\mathrm{c}} + pbc_{\mathrm{c}}^*$. $\square$

### Proofs for Section 4

#### Proof of Proposition 4.1

We prove the claim by contraposition. To this end assume that there exists $x^0\in C$, $x^0\not =0$, such that $c^*(I-A)^{-1}x^0=0$, that is,

$$\begin{aligned} c^*A^tx^0=0\quad \forall \, t\in {\mathbb {Z}}_+\,. \end{aligned}$$

We will show that (1.1) is not ultimately semi-globally persistent. Let $u\in {\mathcal {F}}({\mathbb {Z}}_+,U)$, let $v(t)\equiv 0$ and set $x(t):=x(t;x^0,u,0)$. Then $f(u(0),c^*x^0)=f(u(0),0)=0$ and so, $x(1)=Ax^0$ and $c^*x(1)=c^*Ax^0=0$. A trivial induction argument shows that $x(t)=A^tx^0$ and $c^*x(t)=0$ for all $t\in {\mathbb {Z}}_+$. Since, by exponential stability, $A^tx^0\rightarrow 0$ as $t\rightarrow \infty$, we see that (1.1) is not ultimately semi-globally persistent. $\square$

#### Proof of Proposition 4.3

The proof of statement (a) is trivial. To prove statement (b), assume that *g* is continuous, $g(y)>0$ for $y>0$ and $\limsup _{y\rightarrow \infty }(g(y)/y)<p/u^+$. We focus on the case wherein $f(w,y)=g(wy)$. The other case (*f* given $f(w,y)=wg(y)$) is straightforward and left to the reader. It is clear that *f* is continuous and $f(w,y)>0$ for all $w\in U$ and $y>0$. Moreover, for every $y>0$, there exists $\xi _y\in [u^-y,u^+y]\subset (0,\infty )$ such that

$$\begin{aligned} \max _{w\in U}\frac{g(wy)}{wy}=\frac{g(\xi _y)}{\xi _y}\,. \end{aligned}$$

Now, for $$y>0$$,

$$\begin{aligned} \frac{1}{y}\max _{w\in U}f(w,y)\le \frac{1}{y}\max _{w\in U}\frac{u^+g(wy)}{w} =u^+\max _{w\in U}\frac{g(wy)}{wy}=u^+\frac{g(\xi _y)}{\xi _y}\,, \end{aligned}$$

and so, since $\xi _y\rightarrow \infty$ as $y\rightarrow \infty$,

$$\begin{aligned} \limsup _{y\rightarrow \infty }\frac{1}{y}\max _{w\in U}f(w,y)\le u^+\limsup _{y\rightarrow \infty } \frac{g(\xi _y)}{\xi _y}<p\,, \end{aligned}$$

showing that (N1) holds. Similarly, for every $y>0$, there exists $\zeta _y\in [u^-y, u^+y] \subset (0,\infty )$ such that

$$\begin{aligned} \min _{w\in U}\frac{g(wy)}{wy}=\frac{g(\zeta _y)}{\zeta _y}\,. \end{aligned}$$

Thus, for $$y>0$$,

$$\begin{aligned} \frac{1}{y}\min _{w\in U}f(w,y)\ge \frac{1}{y}\min _{w\in U}\frac{u^-g(wy)}{w} =u^-\min _{w\in U}\frac{g(wy)}{wy}=u^-\frac{g(\zeta _y)}{\zeta _y}\,, \end{aligned}$$

and consequently, since $\zeta _y\rightarrow 0$ as $y\rightarrow 0$,

$$\begin{aligned} \liminf _{y\rightarrow 0}\frac{1}{y}\min _{w\in U}f(w,y)\ge u^-\liminf _{y\rightarrow 0} \frac{g(\zeta _y)}{\zeta _y}>p\,, \end{aligned}$$

establishing that (N2) is satisfied. $\square$

To facilitate the proof of Theorem 4.4, we state and prove two auxiliary results.

#### Lemma A.1

Assume that (L1), (L2) and (P1) are satisfied. The following statements hold.

(a): $$c^*(A+\lambda bc^*)^\tau \in \mathrm{int}\,C^*$$ for every $$\lambda >0$$.
(b): $${{\mathbf {G}}}(1)>0$$, or, equivalently, $$p<\infty $$.
(c): For every $\lambda \in [0,p)$, A.1 $$\begin{aligned} z^*_\lambda :=c^*(I-(A+\lambda bc^*))^{-1}\in \mathrm{int}\,C^*\quad \text{ and }\quad z^*_\lambda (A+pbc^*)=z^*_\lambda \,. \end{aligned}$$ Furthermore, for $\lambda ,\mu \in [0,p)$, A.2 $$\begin{aligned} z^*_\lambda =\frac{p-\mu }{p-\lambda }z^*_\mu . \end{aligned}$$

#### Proof

- The proof is similar to statement (a) of Lemma 4.5, and so is omitted.
- We prove the claim by contraposition. To this end assume that ${{\mathbf {G}}}(1)=0$. We show that (P1) does not hold. Letting $\lambda \in (0,p)$, then, by Proposition 3.1, $A+\lambda bc^*$ is exponentially stable and, therefore, appealing to (2.6), we conclude that
  $$\begin{aligned} \sum _{t=0}^\infty c^*(A+\lambda bc^*)^tb=c^*(I-(A+\lambda bc^*))^{-1}b= \frac{{{\mathbf {G}}}(1)}{1-\lambda {{\mathbf {G}}}(1)}=0. \end{aligned}$$
  Since $c^*(A+\lambda bc^*)^tb\ge 0$ for $t\in {\mathbb {Z}}_+$, it follows that $c^*(A+\lambda bc^*)^tb=0$ for $t\in {\mathbb {Z}}_+$. By (L2), $b\not =0$, and therefore, for all $t\in {\mathbb {Z}}_+$, $c^*(A+\lambda bc^*)^t\not \in \mathrm{int}\,C^*$. Invoking statement (a), we see that (P1) does not hold.
- Let $\lambda \in [0,p)$. By Proposition 3.1, $A+\lambda bc^*$ is exponentially stable, and so $z^*_\lambda$ is well defined. We show first that $z^*_\lambda (A+pbc^*)=z^*_\lambda$. To this end, note that
  $$\begin{aligned} z^*_\lambda (A+pbc^*)= & {} z^*_\lambda (A+\lambda bc^*-I+(p-\lambda )bc^*+I)\\= & {} -c^*+(p-\lambda )z^*_\lambda bc^*+z^*_\lambda \,. \end{aligned}$$
  Using (2.6), we obtain
  $$\begin{aligned} (p-\lambda )z^*_\lambda bc^*=(p-\lambda )c^*(I-(A+\lambda bc^*))^{-1}bc^*= (p-\lambda )\frac{{{\mathbf {G}}}(1)}{1-\lambda {{\mathbf {G}}}(1)}c^*=c^*\,, \end{aligned}$$
  and thus, $$z^*_\lambda (A+pbc^*)=z^*_\lambda $$.
  Next we derive (A.2). Letting $\lambda ,\mu \in [0,p)$, we note that, by the second resolvent identity,
  $$\begin{aligned} z^*_\lambda -z^*_\mu = (\lambda -\mu )c^*(I-(A+\lambda bc^*))^{-1}bc^*(I-(A+\mu bc^*))^{-1}\,, \end{aligned}$$
  and so, using (2.6),
  $$\begin{aligned} z^*_\lambda -z^*_\mu =\frac{(\lambda -\mu ){{\mathbf {G}}}(1)}{1-\lambda {{\mathbf {G}}}(1)}z^*_\mu \,. \end{aligned}$$
  Consequently,
  $$\begin{aligned} z^*_\lambda =\frac{1-\mu {{\mathbf {G}}}(1)}{1-\lambda {{\mathbf {G}}}(1)}z^*_ \mu =\frac{p-\mu }{p-\lambda }z^*_\mu \,, \end{aligned}$$
  establishing (A.2). We proceed to show that $z^*_\lambda \in \mathrm{int}\,C^*$ for $\lambda \in (0,p)$. Since $A+\lambda bc^*$ is exponentially stable, we have
  $$\begin{aligned} z^*_\lambda =\sum _{t=0}^\infty c^*(A+\lambda bc^*)^t\ge c^*(A+\lambda bc^*)^\tau \,, \end{aligned}$$
  and it follows from statement (a) and Lemma 2.1 that $z^*_\lambda \in \mathrm{int}\,C^*$. Finally, an application of (A.2) for $\lambda =0$ and $\mu \in (0,p)$ shows that $z^*_0\in \mathrm{int}\,C^*$. $\square$

To prove Theorem 4.4, it is useful to appeal to the concept of difference inclusions. Indeed, note that solutions of (1.1) are also solutions of a difference inclusion of the form

A.3 $$\begin{aligned} x^\nabla -Ax-v\in bF(c^*x)\,, \end{aligned}$$

where the set-valued function *F* is given by

$$\begin{aligned} F(y):=\big \{f(w,y):w\in U\big \}\quad \forall \, y\ge 0\,. \end{aligned}$$

The following proposition records stability properties of the difference inclusion (A.3).

#### Proposition A.2

Assume that (L1) and (L2) hold and *F* is a set-valued function defined on ${\mathbb {R}}_+$, the values of which are non-empty subsets of ${\mathbb {R}}_+$. Let $p >0$ be as in (3.1). If there exist $\theta \ge 0$ and $q\in (0,p)$ such that $\cup _{0\le y\le \theta }F(y)$ is bounded and

A.4 $$\begin{aligned} \varphi \le qy\quad \forall \,\varphi \in F(y),\,\,\forall \, y\ge \theta , \end{aligned}$$

then there exist $M\ge 1$, $\mu \in (0,1)$ and $N>0$ such that, for every solution $x:{\mathbb {Z}}_+\rightarrow C$ of (A.3),

A.5 $$\begin{aligned} \Vert x(t)\Vert \le M\mu ^t\Vert x(0)\Vert +N(\beta _F+\Vert v\Vert _{\ell ^\infty (0,t)})\quad \forall \, t\in {\mathbb {Z}}_+, \,\,\forall \, v\in {\mathcal {F}}({\mathbb {Z}}_+,C)\,, \end{aligned}$$

where

$$\begin{aligned} \beta _F:=\sup _{0\le y\le \theta ,\,\varphi \in F(y)}\big (\varphi -qy\big )\ge 0\,. \end{aligned}$$

#### Proof

We proceed in two steps.

*Step 1*$$\theta =0$$.

Assume that (A.4) holds with $\theta =0$ (in which case $F(0)=\{0\}$). If the values of *F* are singletons (in which case *F* can be identified with a real-valued function), then the claim is an immediate consequence of Theorem 2.3. It is not difficult to see that Theorem 2.3 extends to set-valued nonlinearities and so, in the case wherein $\theta =0$, the estimate (A.5) follows with $\beta _F=0$.

*Step 2*$$\theta >0$$.

Note that by the boundedness of the set $\cup _{0\le y\le \theta }F(y)$, the constant $\beta _F$ is finite. Define a set-valued map ${\tilde{F}}$ by

$$\begin{aligned} {\tilde{F}}(y):=\left\{ \begin{array}{l l} [0,qy],&{}\,\,\, 0\le y\le \theta ,\\ F(y), &{}\,\,\, y>\theta .\end{array}\right. \end{aligned}$$

and set $e(y):=\max \big (0,\max \{\varphi -qy:\varphi \in F(y)\}\big )$ for $y\in [0,\theta ]$. It is clear that

$$\begin{aligned} \varphi \le qy\quad \forall \,\varphi \in {\tilde{F}}(y),\,\,\forall \,y\ge 0, \end{aligned}$$

and, moreover, $\sup _{0\le y\le \theta }e(y)=\beta _F$. If $x:{\mathbb {Z}}_+\rightarrow C$ is a solution of (A.3), then

$$\begin{aligned} x^\nabla -Ax-v-bd\in b{\tilde{F}}(c^*x)\,, \end{aligned}$$

where $d(t)=0$ if $c^*x(t)>\theta$ and $d(t)\in [0,e(c^*x(t))]$ if $c^*x(t)\le \theta$. Consequently, by Step 1, there exist constants $M\ge 1$, $\mu \in (0,1)$ and $L>0$ such that, for every solution $x:{\mathbb {Z}}_+\rightarrow C$ of (A.3),

$$\begin{aligned} \Vert x(t)\Vert\le & {} M\mu ^t\Vert x(0)\Vert +L\Vert v+bd\Vert _{\ell ^\infty (0,t)}\\ {}\le & {} M\mu ^t\Vert x(0)\Vert +L\Vert v\Vert _{\ell ^\infty (0,t)}+L\beta _F\Vert b\Vert \quad \forall \,t\in {\mathbb {Z}}_+, \end{aligned}$$

where we have used that $|d(t)|=d(t)\le \sup _{0\le y\le \theta }e(y)=\beta _F$. The claim now follows with $N:=L\max (1,\Vert b\Vert )$. $\square$

We are now in the position to prove Theorem 4.4.

#### Proof of Theorem 4.4

- Define a set-valued nonlinearity *F* by
  $$\begin{aligned} F(y):=\big \{f(w,y):w\in U\big \}\,\,\forall \, y\in {\mathbb {R}}_+. \end{aligned}$$
  By (N1), there exist $\theta >0$ and $q\in (0,p)$ such that
  $$\begin{aligned} 0<\varphi \le qy\quad \forall \, y\ge \theta ,\,\,\forall \, \varphi \in F(y). \end{aligned}$$
  It is clear that, for all $x^0\in \Gamma$, $u\in {\mathcal {F}}({\mathbb {Z}}_+,U)$ and all $v\in F({\mathbb {Z}}_+,C_\rho )$, the solution $x(\cdot \,;x^0,u,v)$ is also a solution of the difference inclusion
  $$\begin{aligned} x^{\nabla }-Ax-v\in bF(c^*x)\,, \end{aligned}$$
  and it follows from Proposition A.2 that there exists $\gamma >0$ such that $\Vert x(t;x^0,u,v)\Vert \le \gamma$ for all $x^0\in \Gamma$, $u\in {\mathcal {F}}({\mathbb {Z}}_+,U)$, all $v\in {\mathcal {F}}({\mathbb {Z}}_+,C_\rho )$ and all $t\in {\mathbb {Z}}_+$.
- Let $\Gamma \subset C$ be bounded, closed and such that $0\not \in \Gamma$, and let $\rho >0$. We proceed in two steps.
  *Step 1*: Existence of $\delta$.
  By (N2), there exists $y^\sharp >0$ such that
  A.6 $$\begin{aligned} f(w,y)\ge py\quad \forall \, w\in U,\,\,\forall \, y\in [0,y^\sharp ]\,. \end{aligned}$$
  Let $\gamma >0$ be as in statement (a), and set $y^\dagger :=\max \big \{y^\sharp ,\Vert c^*\Vert \gamma \big \}$ and
  A.7 $$\begin{aligned} \lambda :=\inf \big \{f(w,y)/y:w\in U,\,y^\sharp \le y\le y^\dagger \big \}>0\,. \end{aligned}$$
  Let $x^0\in \Gamma$, $u\in {\mathcal {F}}({\mathbb {Z}}_+,U)$, $v\in {\mathcal {F}}({\mathbb {Z}}_+,C_\rho )$ and write
  $$\begin{aligned} x(t):=x(t;x^0,u,v)\quad \text{ and }\quad y(t):=c^*x(t;x^0,u,v)\qquad \forall \, t\in {\mathbb {Z}}_+\,. \end{aligned}$$
  For given $t\in {\mathbb {Z}}_+$, we have either $y(t)\in [0,y^\sharp ]$ or $y(t)>y^\sharp$.
  Case 1:$y(t)\in [0,y^\sharp ]$. By (A.6), $f(u(t),y(t))\ge py(t)$, and so
  $$\begin{aligned} x(t+1)\ge (A+pbc^*)x(t)\,. \end{aligned}$$
  By assumption (P1) and statement (c) of Lemma A.1 there exists $z^*\in \mathrm{int}\,C^*$ such that $z^* (A+pbc^*)=z^*$, and consequently,
  A.8 $$\begin{aligned} z^*(x(t+1))\ge z^*(x(t))\,. \end{aligned}$$
  Case 2:$y(t)>y^\sharp$. By the definition of $y^\dagger$, $y(t) \le y^\dagger$ and so, invoking (A.7),
  $$\begin{aligned} f(u(t),y(t))\ge \lambda y(t)\ge \lambda y^\sharp \,. \end{aligned}$$
  As a consequence, $x(t+1)\ge Ax(t)+\lambda y^\sharp b$, and so,
  A.9 $$\begin{aligned} z^*(x(t+1))\ge \lambda y^\sharp z^*(b)>0\,. \end{aligned}$$
  Combining (A.8) and (A.9) (the outcomes of the considerations in Cases 1 and 2), we conclude that
  $$\begin{aligned} z^*(x(t+1))\ge \min \{z^*(x(t)),\lambda y^\sharp z^*(b)\} \quad \forall \, t\in {\mathbb {Z}}_+\,, \end{aligned}$$
  whence
  $$\begin{aligned} z^*(x(t))\ge \min \big \{z^*(x^0),\lambda y^\sharp z^*(b) \big \}\ge \varepsilon \quad \forall \, t\in {\mathbb {Z}}_+\,, \end{aligned}$$
  where $\varepsilon >0$ is the minimum of $\inf _{\xi \in \Gamma }z^*(\xi )$ and $\lambda y^\sharp z^*(b)$ (note that the infimum is positive because $\Gamma$ is bounded, closed and $0\not \in \Gamma$). It follows that there exists $\delta >0$ such that $\Vert x(t;x^0,u,v)\Vert \ge \delta$ for all $x^0\in \Gamma$, $u\in {\mathcal {F}}({\mathbb {Z}}_+,U)$, all $v\in {\mathcal {F}}({\mathbb {Z}}_+,C_\rho )$ and all $t\in {\mathbb {Z}}_+$.
  *Step 2* : Existence of $\eta$.
  Setting $\mu :=\min \{\lambda ,p\}>0$ and appealing to (A.6) and (A.7), we obtain
  $$\begin{aligned} x(t+1)\ge (A+\mu bc^*)x(t)\quad \forall \, t\in {\mathbb {Z}}_+, \end{aligned}$$
  and hence, with $\tau$ from (P1),
  $$\begin{aligned} c^*x(t+\tau )\ge c^*(A+\mu bc^*)^\tau x(t)\quad \forall \, t\in {\mathbb {Z}}_+. \end{aligned}$$
  By (P1) and statement (a) of Lemma A.1, $c^*(A+\mu bc^*)^\tau \in \mathrm{int}\,C^*$. Now, $\Vert x(t)\Vert \ge \delta >0$ for all $t\in {\mathbb {Z}}_+$, and thus, invoking Lemma 2.1, there exists $\eta >0$ such that $c^*x(t+\tau )\ge \eta$ for all $x^0\in \Gamma$, $u\in {\mathcal {F}}({\mathbb {Z}}_+,U)$, $v\in {\mathcal {F}}({\mathbb {Z}}_+,C_\rho )$ and all $t\in {\mathbb {Z}}_+$, completing the proof. $\square$

#### Proof of Lemma 4.5

- Assume that (P2) holds and let $\lambda \in (0,1)$. A routine induction argument shows that, for every $t\in {\mathbb {Z}}_+$,
  $$\begin{aligned} c^*(A+\lambda bc^*)^t\ge c^*(A+bc^*)^t\quad \text{ if } \lambda \ge 1, \end{aligned}$$
  and
  $$\begin{aligned} c^*(A+\lambda bc^*)^t\ge \lambda ^tc^*(A+bc^*)^t\quad \text{ if } 0<\lambda <1. \end{aligned}$$
  Combined, the above inequalities yield that
  A.10 $$\begin{aligned} c^*(A+\lambda bc^*)^t\ge \min (\lambda ^t,1)c^*(A+bc^*)^t\quad \forall \, t\in {\mathbb {Z}}_+\,. \end{aligned}$$
  Invoking (A.10), we conclude that
  $$\begin{aligned} c^*\sum _{t=0}^\tau (A+\lambda bc^*)^t\ge \lambda ^\tau c^*\sum _{t=0}^\tau (A+bc^*)^t\,. \end{aligned}$$
  Hence, setting $A_\lambda :=A+\lambda bc^*$, it follows from Lemma 2.1 that
  A.11 $$\begin{aligned} c^*\sum _{t=0}^\tau A^t_\lambda \in \mathrm{int}\,C^*\,. \end{aligned}$$
  Now $c^*(A+bc^*)^\tau =c^*(A_\lambda +(1-\lambda )bc^*)^\tau$ and a straightforward induction argument shows that
  A.12 $$\begin{aligned} c^*(A+bc^*)^\tau \ge c^*\sum _{t=0}^\tau [(1-\lambda )(c^*b)]^{\tau -t}A^t_\lambda \ge \alpha (\tau )c^*\sum _{t=0}^\tau A^t_\lambda \,, \end{aligned}$$
  where
  $$\begin{aligned} \alpha (\tau ):=\left\{ \begin{array}{l l} [(1-\lambda )(c^*b)]^\tau ,&{}\,\,\,\text {if }(1-\lambda )c^*b< 1\\ 1, &{}\,\,\,\text {if } (1-\lambda )c^*b\ge 1.\end{array}\right. \end{aligned}$$
  By hypothesis, $\alpha (\tau )>0$, and so, invoking (A.11), (A.12) and Lemma 2.1, we obtain that $c^*(A+bc^*)^\tau \in \mathrm{int}\,C^*$, establishing that (P2) implies (P1). If (P3) holds, then $c^*(A+bc^*)^\tau =[(A+bc^*)^*]^\tau c^*\in \mathrm{int}\,C^*$, showing that (P1) is satisfied.
- It is sufficient to show that (P3) holds. Since $c^*b>0$, it is clear that the matrix $bc^*$ has positive trace and so the trace of $A+bc^*$ is also positive. It is well known that irreducible non-negative matrices with positive trace are primitive (see, for example, Meyer (2000), Example 8.3.3), and consequently, (P3) is satisfied.
- Let $\lambda >0$. A routine argument similar to that in the proof of statement (a) shows that
  A.13 $$\begin{aligned}{}[(A+\lambda bc^*)^*]^t x^* \ge \min (\lambda ^t,1) [(A+bc^*)^*]^t x^* \quad \forall \, t\in {\mathbb {Z}}_+, \; \forall \, x^* \in C^*\,. \end{aligned}$$
  Assume that $\tau \in {\mathbb {Z}}_+$ is such that $[(A+bc^*)^*]^\tau$ is strictly positive. Then, in light of (A.13), an application of statement (b) of Lemma 2.1 shows that $[(A+\lambda bc^*)^*]^\tau x^*\in \mathrm{int}\,C^*$ for all non-zero $x^* \in C^*$. $\square$

### Proofs for Section 5

#### Proof of Lemma 5.1

Since $x^{\mathrm{e}}=py^{\mathrm{e}}\sum _{k=0}^\infty A^kb$, assumptions (L1) and (L2) imply that $x^{\mathrm{e}}\ge 0$. It follows immediately from the definitions of $x^{\mathrm{e}}$ and *p* that $c^*x^{\mathrm{e}}=y^{\mathrm{e}}$. Consequently, since $y^{\mathrm{e}}>0$, we conclude that $x^{\mathrm{e}}\not = 0$, and so $x^{\mathrm{e}}>0$. To show that $x^{\mathrm{e}}$ is an equilibrium, note that

$$\begin{aligned} x^{\mathrm{e}}=(I-A)^{-1}bpy^{\mathrm{e}}=(I-A)^{-1}bf(u^{\mathrm{e}},y^{\mathrm{e}}) =(I-A)^{-1}bf(u^{\mathrm{e}},c^*x^{\mathrm{e}}), \end{aligned}$$

and so, $x^{\mathrm{e}}=Ax^{\mathrm{e}}+bf(u^{\mathrm{e}},c^*x^{\mathrm{e}})$. As for uniqueness, let $x^\dagger$ be an another non-zero vector in *C* satisfying

A.14 $$\begin{aligned} x^\dagger =Ax^\dagger +bf(u^{\mathrm{e}},c^*x^\dagger ). \end{aligned}$$

Then $c^*x^\dagger ={{\mathbf {G}}}(1)f(u^{\mathrm{e}},c^*x^\dagger )$, and thus, $f(u^{\mathrm{e}},c^*x^\dagger )=pc^*x^\dagger$. Now $c^*x^\dagger \not =0$ (otherwise, due to (A.14), 1 would be an eigenvalue of *A* which is not possible by (L1)), and so, since $y^{\mathrm{e}}$ is the unique positive solution of the equation $f(u^{\mathrm{e}},y)=py$, we see that $c^*x^\dagger =y^{\mathrm{e}}=c^*x^{\mathrm{e}}$, whence

$$\begin{aligned} x^\dagger =(I-A)^{-1}bf(u^{\mathrm{e}},c^*x^\dagger )=(I-A)^{-1}bpy^{\mathrm{e}}=x^{\mathrm{e}}. \end{aligned}$$

Let us now additionally assume that (P3) holds. Noting that

$$\begin{aligned} (A+pbc^*)x^{\mathrm{e}}=x^{\mathrm{e}}+(A-I+pbc^*)x^{\mathrm{e}}=x^{\mathrm{e}}-pby^{\mathrm{e}}+pby^{\mathrm{e}} =x^{\mathrm{e}}, \end{aligned}$$

we see that $(A+pbc^*)^\tau x^{\mathrm{e}}=x^{\mathrm{e}}$. Consequently, for every $x^*\in C^*$, $x^*\not =0$,

$$\begin{aligned} x^*(x^{\mathrm{e}})=x^*((A+pbc^*)^\tau x^{\mathrm{e}})=\big ([(A+pbc^*)^*]^\tau x^*\big )(x^{\mathrm{e}})>0. \end{aligned}$$

The inequality holds because $x^{\mathrm{e}}>0$ and $[(A+pbc^*)^*]^\tau x^*\in \mathrm{int}\,C^*$ (as follows from (P3) and Lemma 4.5). $\square$

#### Proof of Theorem 5.2

- By statement (a) of Theorem 4.4, there exists $\gamma >0$ such that, for all $x^0\in \Gamma$, $u\in {\mathcal {F}}({\mathbb {Z}}_+,U)$ and $v\in {\mathcal {F}}({\mathbb {Z}}_+,C_\rho )$,
  $$\begin{aligned} \Vert x(t;x^0,u,v)\Vert \le \gamma \quad \forall \, t\in {\mathbb {Z}}_+. \end{aligned}$$
  Now let $x^0\in \Gamma$, $u\in {\mathcal {F}}({\mathbb {Z}}_+,U)$ and $v\in {\mathcal {F}}({\mathbb {Z}}_+,C_\rho )$ be fixed, but arbitrary, set $h(y):=f(u^{\mathrm{e}},y)$, and write $x(t):=x(t;x^0,u,v)$. Then
  $$\begin{aligned} x^\nabla =Ax+b(h(c^*x)+d)+v,\quad \text{ where } d:=f(u,c^*x)- f(u^{\mathrm{e}},c^*x). \end{aligned}$$
  Since $c^*x^{\mathrm{e}}=y^{\mathrm{e}}$ and $x^{\mathrm{e}}=Ax^{\mathrm{e}}+bh(y^{\mathrm{e}})$, it follows that the function ${\tilde{x}}(t):=x(t)-x^{\mathrm{e}}$ satisfies
  A.15 $$\begin{aligned} {\tilde{x}}(t+1)=A{\tilde{x}}(t)+b\big [h(c^*{\tilde{x}}(t)+y^{\mathrm{e}})-h(y^{\mathrm{e}})\big ]+ bd(t)+v(t),\quad \forall \, t\in {\mathbb {Z}}_+. \end{aligned}$$
  By the hypothesis of ultimate semi-global $c^*$-persistence, there exist $\tau \in {\mathbb {Z}}_+$ and $\eta \in (0,y^{\mathrm{e}})$ such that
  A.16 $$\begin{aligned} c^*{\tilde{x}}(t+\tau )\ge -y^{\mathrm{e}}+\eta \quad \forall \, t\in {\mathbb {Z}}_+. \end{aligned}$$
  Defining
  $$\begin{aligned} {\tilde{h}}:{\mathbb {R}}\rightarrow {\mathbb {R}},\,\,\, y\mapsto \left\{ \begin{array}{l l} h(y+y^{\mathrm{e}})-h(y^{\mathrm{e}}),&{}\quad \text {for }y\ge -y^{\mathrm{e}}+\eta \\ h(\eta )-h(y^{\mathrm{e}}), &{}\quad \text {for }y<-y^{\mathrm{e}}+\eta ,\end{array}\right. \end{aligned}$$
  it follows from (A.15) and (A.16) that
  A.17 $$\begin{aligned} {\tilde{x}}(t+1)=A{\tilde{x}}(t)+b\big [{\tilde{h}}(c^*{\tilde{x}}(t))+d(t)\big ]+v(t), \quad \forall \, t\in \tau +{\mathbb {Z}}_+. \end{aligned}$$
  By assumption (N3), there exists $q\in (0,p)$ such that (5.3) holds and consequently,
  A.18 $$\begin{aligned} |{\tilde{h}}(y)|\le q|y|\,\,\,\forall \, y\in {\mathbb {R}}. \end{aligned}$$
  Invoking (L1) and (L2), it follows from Proposition 3.1 that ${{\mathbf {G}}}(1)=\Vert {{\mathbf {G}}}\Vert _{H^\infty }$, implying that $p=1/\Vert {{\mathbf {G}}}\Vert _{H^\infty }$. Combining this with (A.18) and appealing to Theorem 2.3, we conclude that system (A.17) is exponentially ISS (with respect to the forcing $bd+v$). Hence, there exist $M_0\ge 1$, $\mu \in (0,1)$ and $N_0>0$ (depending only on *A*, *b*, $c^*$, $\rho$, *U*, *f* and $\Gamma$) such that
  A.19 $$\begin{aligned} \Vert {\tilde{x}}(t+\tau )\Vert \le M_0\mu ^t\Vert {\tilde{x}}(\tau )\Vert +N_0( \Vert d\Vert _{\ell ^\infty (0,t)}+\Vert v\Vert _{\ell ^\infty (0,t)})\quad \forall \, t\in {\mathbb {Z}}_+. \end{aligned}$$
  Furthermore, since the function $y\mapsto h(y+y^{\mathrm{e}})-h(y^{\mathrm{e}})$ is linearly bounded on $[-y^{\mathrm{e}},\infty )$, we obtain from (A.15) that
  A.20 $$\begin{aligned} \Vert {\tilde{x}}(t)\Vert \le M_1\Vert {\tilde{x}}(0)\Vert + N_1\big (\Vert d\Vert _{\ell ^\infty (0,t)}+\Vert v\Vert _{\ell ^\infty (0,t)}\big ) \quad t=1,2,\ldots ,\tau , \end{aligned}$$
  where the positive constants $M_1$ and $N_1$ depend only on *A*, *b*, $c^*$ and *f*. Setting $M:=\max (M_0,M_1\mu ^{-\tau })$ and $N:=\max (N_0,N_1)$ and appealing to (A.19) and (A.20), we arrive at
  $$\begin{aligned} \Vert {\tilde{x}}(t)\Vert \le M\mu ^t\Vert {\tilde{x}}(0)\Vert +N(\Vert d\Vert _{\ell ^\infty (0,t)}+ \Vert v\Vert _{\ell ^\infty (0,t)})\quad \forall \, t\in {\mathbb {Z}}_+\,. \end{aligned}$$
  Combining this with
  $$\begin{aligned} \Vert d\Vert _{\ell ^\infty (0,t)}\le & {} \max \big \{|f(u^{\mathrm{e}},y)-f(u(s),y)|:s=0,1,\ldots ,t,\,\, 0\le y\le \gamma \Vert c^*\Vert \big \}\\&=\beta (u,t)\,, \end{aligned}$$
  shows that (5.5) holds, completing the proof of statement (1).
- Let $x^0\in C$ with $x^0\not =0$, let $u\in {\mathcal {F}}({\mathbb {Z}}_+,U)$ and $v\in {\mathcal {F}}({\mathbb {Z}}_+,C)$ be such that $\lim _{t\rightarrow \infty }u(t)=u^{\mathrm{e}}$ and $\lim _{t\rightarrow \infty }v(t)=0$. Obviously, there exists $\rho >0$ such that $v\in {\mathcal {F}}({\mathbb {Z}}_+,C_\rho )$, and, by Theorem 4.4, there exists a bounded closed set $K\subset C$ such that $0\not \in K$ and $x(t;x^0,u,v)\in K$ for $t\in {\mathbb {Z}}_+$. By statement (a) (with *K* now playing the role of $\Gamma$), there exist $M\ge 1$, $\mu \in (0,1)$ and $N>0$ such that, for all $s,t\in {\mathbb {Z}}_+$,
  $$\begin{aligned} \Vert x(t;x(s;x^0,u,v),u_s,v_s)-x^{\mathrm{e}}\Vert\le & {} M\mu ^t\Vert x(s;x^0,u,v)-x^{\mathrm{e}}\Vert \\&+\,N(\beta (u_s,t)+\Vert v_s\Vert _{\ell ^\infty })\,, \end{aligned}$$
  where $u_s(t)=u(t+s)$ and $v_s(t)=v(t+s)$ for all $t\in {\mathbb {Z}}_+$.
  Now $x(t+s;x^0,u,v)=x(t;x(s;x^0,u,v),u_s,v_s)$ and so, for all $s,t\in {\mathbb {Z}}_+$,
  $$\begin{aligned} \Vert x(t+s;x^0,u,v)-x^{\mathrm{e}}\Vert \le M\mu ^t\Vert x(s;x^0,u,v)-x^{\mathrm{e}}\Vert + N(\beta (u_s,t)+\Vert v_s\Vert _{\ell ^\infty })\,. \end{aligned}$$
  Given $\varepsilon >0$, there exists $\sigma \in {\mathbb {Z}}_+$ such that $N(\beta (u_{\sigma },t)+\Vert v_\sigma \Vert _{\ell ^\infty })\le \varepsilon /2$ for all $t\in {\mathbb {Z}}_+$ (where we have used that $\sup _{t\in {\mathbb {Z}}_+}\beta (u_s,t)+\Vert v_s\Vert _{\ell ^\infty }\rightarrow 0$ as $s\rightarrow \infty$). Moreover, choose $\theta \in {\mathbb {Z}}_+$ such that $M\mu ^t\Vert x(\sigma ;x^0,u,v)-x^{\mathrm{e}}\Vert \le \varepsilon /2$ for all $t\ge \theta$. Consequently,
  $$\begin{aligned} \Vert x(t;x^0,u,v)-x^{\mathrm{e}}\Vert \le \varepsilon \quad \forall \, t\in (\sigma +\theta )+{\mathbb {Z}}_+\,, \end{aligned}$$
  completing the proof. $\square$

#### Proof of Corollary 5.6

Let $x^0\in C$, $x^0\not =0$ and $u^\infty >0$. Consider fixed, but arbitrary elements $u\in {\mathcal {F}}({\mathbb {Z}}_+,(0,\infty ))$ and $v\in {\mathcal {F}}({\mathbb {Z}}_+,C)$ such that $u(t)\rightarrow u^\infty$ and $v(t)\rightarrow 0$ as $t\rightarrow \infty$. The strategy is to apply statement (b) of Theorem 5.2. To this end, let *U* be any compact set in ${\mathbb {R}}_+$ with $0\not \in U$, $u^{\mathrm{e}}\in U$ and $(u^{\mathrm{e}}/u^\infty )u(t)\in U$ for all $t\in {\mathbb {Z}}_+$. Setting $f_0:=\lim _{y\rightarrow 0}g(y)y$ and

$$\begin{aligned} f(w,y)=\left\{ \begin{array}{l l} g(wy)y,&{}\quad \text {for }w\in U \text { and }y>0 \\ f_0/w, &{}\quad \text {for } w\in U\text { and }y=0,\end{array}\right. \end{aligned}$$

it follows from (5.6) and Proposition 4.3 that *f* satisfies (N2). The conditions (5.7) and (5.8) imply that (5.1) and (5.2) hold for *f*. Consequently, *f* satisfies (N3). It follows that the hypotheses of Theorem 5.2 hold. Defining, for all $t\in {\mathbb {Z}}_+$,

$$\begin{aligned} {\tilde{x}}(t):=\frac{u^\infty }{u^{\mathrm{e}}}x(t;x^0,u,v),\,\,\, {\tilde{u}}(t):=\frac{u^{\mathrm{e}}}{u^\infty }u(t),\,\,\, {\tilde{v}}(t):=\frac{u^\infty }{u^{\mathrm{e}}}v(t), \end{aligned}$$

we have that ${\tilde{x}}^\nabla =A{\tilde{x}}+bf({\tilde{u}},c^*{\tilde{x}})+{\tilde{v}}$. Finally, ${\tilde{u}}(t)\rightarrow u^{\mathrm{e}}$ and ${\tilde{v}}(t)\rightarrow 0$ as $t\rightarrow \infty$, and hence, an application of statement (b) of Theorem 5.2 shows that ${\tilde{x}}(t)\rightarrow x^{\mathrm{e}}$ as $t\rightarrow \infty$ and so, $x(t;x^0,u,v)\rightarrow (u^{\mathrm{e}}/u^\infty )x^{\mathrm{e}}$ as $t\rightarrow \infty$. $\square$

## An example relating to Rebarber et al. (2012), Corollary 4.1

Here we show, by providing a counter example, that the IPM result (Rebarber et al. 2012, Corollary 4.1) is not correct. To see this, assume that, in the notation of Rebarber et al. (2012), $\Omega =[0,1]$ and the functions $b,c:[0,1]\rightarrow {\mathbb {R}}_+$ and $p:[0,1]\times [0,1]\rightarrow {\mathbb {R}}_+$ are continuous and satisfy

$$\begin{aligned} c(\xi )=0 \quad \forall \, \xi \in [1/2,1]\quad \text{ and }\quad \Vert c\Vert _{L^\infty }>0, \end{aligned}$$

and

$$\begin{aligned} p(x,\xi )=0 \quad \forall \, (x,\xi )\in [0,1/2]\times [1/2,1]\quad \text{ and }\quad \max _{0\le \xi \le 1}\int _0^1p(x,\xi )dx<1. \end{aligned}$$

Then, with $n(\xi ,0)=0$ for $\xi \in [0,1/2]$ and $\Vert n(\cdot \,,0)\Vert _{L^1}>0$, we have that

$$\begin{aligned} c^Tn(\cdot \,,0)=\int _0^1c(\xi )n(\xi ,0)d\xi =0, \end{aligned}$$

and

$$\begin{aligned} \int _0^1p(x,\xi )n(\xi ,0)d\xi = \int _{1/2}^1p(x,\xi )n(\xi ,0)d\xi =0\quad \forall \, x\in [0,1/2]. \end{aligned}$$

Now

$$\begin{aligned} n(x,1)= & {} \int _0^1p(x,\xi )n(\xi ,0)d\xi +b(x)f(c^Tn(\cdot ,0))\\= & {} \int _0^1p(x,\xi )n(\xi ,0)d\xi =0\quad \forall \, x\in [0,1/2], \end{aligned}$$

where we have assumed that $f(0)=0$. By repeated application of the above argument, it follows that

B.1 $$\begin{aligned} y(t)=c^Tn(\cdot \,,t)=0\quad \forall \, t\in {\mathbb {Z}}_+. \end{aligned}$$

If we choose *b* so that additionally $c^Tb=\int _0^1c(\xi )b(\xi )d\xi >0$, it is clear that $c^T(I-A)^{-1}b>0$, and so for any non-decreasing concave function *f* with $f(0)=0$, $\lim _{y\rightarrow 0}f(y)/y>p^*_e$ and $\lim _{y\rightarrow \infty }f(y)/y<p^*_e$, the assumptions of Rebarber et al. (2012), Corollary 4.1 are satisfied, where $1/p^*_e=c^T(I-A)^{-1}b$. But if Rebarber et al. (2012), Corollary 4.1 was correct, then $\lim _{t\rightarrow \infty }y(t)>0$, in contradiction to (B.1).

Finally, denoting the integral operator

$$\begin{aligned} L^1(0,1)\rightarrow L^1(0,1),\,\,\zeta \mapsto \int _0^1 p(\cdot \,,\xi )\zeta (\xi )d\xi \end{aligned}$$

by *A*, an inspection of the above argument shows that $\ker c^T$ has an *A*-invariant subspace $S\subset L^1(0,1)$ containing positive elements.

## Example 6.4: further details

We provide some details on the numerical approximation scheme. For notational convenience in this section we set $\underline{N}:= \{ 1,2, \dots ,N\}$ for each $N \in {\mathbb {N}}$.

To derive a numerical approximation of the forced nonlinear IPM (6.24), we discretise the state variable *n* via finite elements. To this end, we consider $n(t+1, \xi )$, multiply by $\psi \in L^1(\Omega ):=L^1(\Omega ,{\mathbb {R}})$ and integrate over $\Omega$ to obtain

C.1 $$\begin{aligned} \int _\Omega \psi (\xi ) n(t+1, \xi ) \, d \xi&= \int _\Omega \psi (\xi ) \Big [\big (A_0 n(t,\cdot \,)\big )(\xi ) + p_{\mathrm{sc}}(u(t)D(t)) D(t) f_{\mathrm{sd}}(\xi ) \Big ]\, d \xi \nonumber \\&= \int _\Omega \psi (\xi ) \left( \int _{\zeta \in \Omega } k(\xi , \zeta ) n(t, \zeta ) \, d\zeta \right) d \xi \nonumber \\&\quad + \left( \int _\Omega \psi (\xi ) f_{\mathrm{sd}}(\xi ) \, d \xi \right) p_{\mathrm{sc}}(u(t)D(t)) D(t)\,. \end{aligned}$$

We seek an approximate solution to (C.1) of the form

C.2 $$\begin{aligned} n_N(t, \xi ) = \sum _{j = 1}^N a_j(t) \phi _j(\xi ), \quad \forall \, t \in {\mathbb {Z}}_+ , \quad \forall \, \xi \in \Omega \,, \end{aligned}$$

where $N \in {\mathbb {N}}$, $\phi _j$ are given functions in $L^1(\Omega )$ and $a_j$ are to-be-determined scalar coefficients. Substituting (C.2) into (C.1), and testing against $\psi = \phi _i$ for each $i \in \underline{N}$ gives

C.3 $$\begin{aligned} \sum _{j = 0}^N \left( \int _\Omega \phi _i(\xi ) \phi _j(\xi ) \, d \xi \right) a_j(t+1)&= \sum _{j=0}^N \left( \int _\Omega \phi _i(\xi ) \int _{\zeta \in \Omega } k(\xi , \zeta ) \phi _j(\xi ) \, d\zeta d \xi \right) a_j(t) \nonumber \\&\quad + \left( \int _\Omega \phi _i(\xi ) f_{\mathrm{sd}}(\xi ) \, d \xi \right) p_{\mathrm{sc}}(u(t)D(t)) D(t) \,. \end{aligned}$$

Setting

$$\begin{aligned} z(t) := \begin{pmatrix} a_1(t)&\dots&a_N(t) \end{pmatrix}^T \in {\mathbb {R}}^{N} \quad \forall \, t \in {\mathbb {Z}}_+\,, \end{aligned}$$

we see that (C.3) may be expressed in matrix form as

C.4 $$\begin{aligned} E z^\nabla = F z + G f(u,D) \,. \end{aligned}$$

Here $(E,F,G) \in {\mathbb {R}}^{N\times N}\times {\mathbb {R}}^{N\times N}\times {\mathbb {R}}^{N}$ are given entrywise by

C.5 $$\begin{aligned} E_{ij} := \int _\Omega \phi _i(\xi ) \phi _j(\xi )d \xi ,\quad F_{ij} := \int _\Omega \phi _i(\xi ) \left( \int _{\Omega } k(\xi , \zeta ) \phi _j(\zeta ) d\zeta \right) d \xi , \quad \forall \,i,j \in \underline{N}\,, \end{aligned}$$

and

C.6 $$\begin{aligned} G_i := \int _\Omega \phi _i(\xi ) f_{\mathrm{sd}}(\xi )d \xi , \quad \forall \,i\in \underline{N}. \end{aligned}$$

Since we are seeking to approximate the $L^1(\Omega )$ functions $n(t, \cdot )$ in $L^1(\Omega )$ (that is, we are not approximating any derivatives), we choose as finite-dimensional approximation spaces the linear span of *N* piecewise constant functions. Specifically, for fixed $N \in {\mathbb {N}}$, we define $\xi _j := m_1 + j(m_2-m_1)/N$ for $j \in \{0,1,\dots ,N\}$, $\Delta := (m_2 - m_1)/N$ and

$$\begin{aligned} \phi _i : \Omega \rightarrow {\mathbb {R}}_+, \quad \phi _i(\xi ) : = \left\{ \begin{aligned}&\frac{1}{\sqrt{\Delta }}&\xi _{i-1} \le \xi \le \xi _i \\&0&\text {else,}\end{aligned} \right. \qquad \forall \,i \in \underline{N}. \end{aligned}$$

An advantage of such a choice is that, as readily seen, $E = I$, because $E_{ij} =0$ if $i \ne j$ and

$$\begin{aligned} E_{ii} = \int _{m_1}^{m_2} \phi ^2_i(\xi ) \, d\xi = \frac{1}{\Delta } \int _{\xi _{i-1}}^{\xi _i} d\xi = 1, \quad \forall \, i \in \underline{N}\,. \end{aligned}$$

Moreover, by inspection of (C.5) and (C.6), it follows that with the above choice of piecewise constant $\phi _j$, *F* and *G* in (C.6) have nonnegative entries.

Noting that

$$\begin{aligned} h^* n_N(t , \cdot )&= \sum _{j=0}^N \left( \int _{\Omega } p_{\mathrm{f}}(\xi ) f_{\mathrm{s}}(\xi ) p_{\mathrm{s}}(\xi ) \phi _j(\xi )\, d \xi \right) a_j(t) \\&= H^T z(t), \quad \forall \, t \in {\mathbb {Z}}_+ \,, \end{aligned}$$

where the entries of $H \in {\mathbb {R}}^N$ are given by

$$\begin{aligned} H_j:= \int _{\Omega } p_{\mathrm{f}}(\xi ) f_{\mathrm{s}}(\xi ) p_{\mathrm{s}}(\xi )\phi _j(\xi )\, d \xi , \quad \forall \, j \in \underline{N}\,, \end{aligned}$$

we arrive at the finite-dimensional approximation to (6.24) given by

C.7 $$\begin{aligned} x_N^\nabla&= \begin{pmatrix} z^\nabla \\ D^\nabla \\ S_1^\nabla \end{pmatrix} = \begin{pmatrix} F &{} 0 &{} 0 \\ p_{\mathrm{est}} g_0 H^T &{} 0 &{} p_{\mathrm{est}}g_1 \\ (1-g_0) s_0 H^T &{} 0 &{} 0 \end{pmatrix}\begin{pmatrix} z \\ D \\ S_1 \end{pmatrix} + \begin{pmatrix} G\\ 0 \\ 0 \end{pmatrix}p_{\mathrm{sc}}\big ( u D) D + v \,. \end{aligned}$$

Note that *F*, *G* and *H* have nonnegative entries. The numerical simulations for Example 6.4 have been generated via (C.7) with $N=30$.

## Footnotes

1 Note that if $\alpha =1$, then $f(w,y)=g(y/w)$, where $g(y)=y/(1+y)$.

2 In this example, we have identified a row vector with the linear functional induced by the row vector. We will continue to do so in the rest of the paper in the context of finite-dimensional examples.

3 Note that Hesse et al. (2008) uses *C* where we use *D* here. This is done as to avoid a clash with our standing notation of *C* denoting a cone in the state space.

4 The model in Hesse et al. (2008) contains an additional stage-class, namely the number of 2-year old seeds $S_2$. But since the parameter $g_2 =0$ [see Hesse et al. (2008), Table 1], $S_2$ does not enter into the other three equations, and so does not play a role in the dynamics and therefore this stage class has been omitted here.

## References

[CR1] Begon M, Mortimer M, Thompson DJ (2009). Population ecology: a unified study of animals and plants.

[CR2] Berman A, Neumann M, Stern RJ (1989). Nonnegative matrices in dynamic systems.

[CR3] Berman A, Plemmons RJ (1994). Nonnegative matrices in the mathematical sciences.

[CR4] Bill A, Guiver C, Logemann H, Townley S (2016). Stability of non-negative Lur’e systems. SIAM J Control Optim.

[CR5] Caswell H (2001). Matrix population models: construction, analysis, and interpretation.

[CR6] Childs DZ, Rees M, Rose KE, Grubb PJ, Ellner SP (2003). Evolution of complex flowering strategies: an age- and size-structured integral projection model. Proc R Soc B-Bio Sci.

[CR7] Chow Y, Jang SR-J, Yeh N-S (2018). Dynamics of a population in two patches with dispersal. J Differ Equ Appl.

[CR8] Coughlan JJ (2007) Absolute stability results for infinite-dimensional discrete-time systems with applications to sampled-data integral control. Ph.D. Thesis, University of Bath

[CR9] Cushing JM (1998). An introduction to structured population dynamics.

[CR10] Dashkovskiy SN, Efimov DV, Sontag ED (2011). Input-to-state stability and allied system properties. Autom Remote Control.

[CR11] Deimling K (1985). Nonlinear functional analysis.

[CR12] Eager EA (2016). Modelling and analysis of population dynamics using Lur’e systems accounting for competition from adult conspecifics. Lett Biomath.

[CR13] Eager EA, Rebarber R (2016). Sensitivity and elasticity analysis of a Lur’e system used to model a population subject to density-dependent reproduction. Math Biosci.

[CR14] Eager EA, Guiver C, Hodgson D, Rebarber R, Stott I, Townley S (2014). Bounds on the dynamics of sink populations with noisy immigration. Theor Popul Biol.

[CR15] Eager EA, Rebarber R, Tenhumberg B (2014). Global asymptotic stability of plant-seed bank models. J Math Biol.

[CR16] Easterling MR, Ellner SP, Dixon PM (2000). Size-specific sensitivity: applying a new structured population model. Ecology.

[CR17] Ellner SP, Rees M (2006). Integral projection models for species with complex demography. Am Nat.

[CR18] Franco D, Logemann H, Perán J (2014). Global stability of an age-structured population model. Syst Control Lett.

[CR19] Franco D, Guiver C, Logemann H, Perán J (2017). Semi-global persistence and stability for a class of forced discrete-time population models. Phys D.

[CR20] Freedman HI, So JW-H (1989). Persistence in discrete semidynamical systems. SIAM J Math Anal.

[CR21] Gilmore M, Guiver C, Logemann H (2019) Stability and convergence properties of infinite-dimensional discrete-time Lur’e system. Int J Control 10.1080/00207179.2019.1575528

[CR22] Haddad WM, Chellaboina V (2008). Nonlinear dynamical systems and control.

[CR23] Haddad WM, Chellaboina V, Hui Q (2010). Nonnegative and compartmental dynamical systems.

[CR24] Hastings A (2004). Transients: the key to long-term ecological understanding?. Trends Ecol Evol.

[CR25] Hesse E, Rees M, Müller-Schärer H (2008). Life-history variation in contrasting habitats: flowering decisions in a clonal perennial herb (*Veratrum album*). Am Nat.

[CR26] Hinrichsen D, Pritchard AJ (2005). Mathematical systems theory I.

[CR27] Jayawardhana B, Logemann H, Ryan EP (2011). The circle criterion and input-to-state stability: new perspectives on a classical result. IEEE Control Syst Mag.

[CR28] Jiang ZP, Wang Y (2001). Input-to-state stability for discrete-time nonlinear systems. Automatica.

[CR29] Karafyllis I, Jiang ZP (2011). Stability and stabilization of nonlinear systems.

[CR30] Khalil HK (2014). Nonlinear systems.

[CR31] Krasnosel’skij MA, Lifshits JA, Sobolev AV (1989). Positive linear systems: the method of positive operators.

[CR32] Krause U (2015). Positive dynamical systems in discrete time.

[CR33] Lax PD (2002). Functional analysis.

[CR34] Liberzon MR (2006). Essays on the absolute stability theory. Autom Remote Control.

[CR35] Luenberger DG (1979). Introduction to dynamic systems: theory, models, and applications.

[CR36] MacDonald N, Watkinson AR (1981). Models of an annual plant population with a seedbank. J Theor Biol.

[CR37] Merow C, Dahlgren JP, Metcalf CJE, Childs DZ, Evans ME, Jongejans E, Record S, Rees M, Salguero-Gómez R, McMahon SM (2014). Advancing population ecology with integral projection models: a practical guide. Methods Ecol Evol.

[CR38] Meyer CD (2000). Matrix analysis and applied linear algebra.

[CR39] Newman TJ, Antonovics J, Wilbur HM (2002). Population dynamics with a refuge: fractal basins and the suppression of chaos. Theor Popul Biol.

[CR40] Rebarber R, Tenhumberg B, Townley S (2012). Global asymptotic stability density dependent integral population projection models. Theor Popul Biol.

[CR41] Sarkans E, Logemann H (2015). Input-to-state stability for Lur’e systems. Math Control Signals Syst.

[CR42] Sarkans E, Logemann H (2016). Input-to-state stability for discrete-time Lur’e systems. SIAM J Control Optim.

[CR43] Sarkans E, Logemann H (2016). Stability of higher-order discrete-time Lur’e systems. Linear Algebra Appl.

[CR44] Schreiber SJ (2012). Persistence for stochastic difference equations: a mini-review. J Differ Equ Appl.

[CR45] Smith HL, Thieme HR (2011). Dynamical systems and population persistence.

[CR46] Smith HL, Thieme HR (2013). Persistence and global stability for a class of discrete-time structured population models. Discrete Contin Dyn-A.

[CR47] Sontag ED (1989). Smooth stabilization implies coprime factorization. IEEE Trans Autom Control.

[CR48] Sontag ED, Nistri P, Stefani G (2006). Input-to-state stability: basic concepts and results. Nonlinear and optimal control theory.

[CR49] Sontag ED (2013). Mathematical control theory: deterministic finite dimensional systems.

[CR50] Staffans OJ (2005). Well-posed linear systems.

[CR51] Stott I, Townley S, Hodgson D (2011). A framework for studying transient dynamics of population projection matrix models. Ecol Lett.

[CR52] Taylor CM, Hastings A (2005). Allee effects in biological invasions. Ecol Lett.

[CR53] Teel AR, Hespanha J (2004). Examples of GES systems that can be driven to infinity by arbitrarily small additive decaying exponentials. IEEE Trans Autom Control.

[CR54] Thompson K, Grime JP (1979). Seasonal variation in the seed banks of herbaceous species in ten contrasting habitats. J Ecol.

[CR55] Townley S, Rebarber R, Tenhumberg B (2012). Feedback control systems analysis of density dependent population dynamics. Syst Control Lett.

[CR56] Vidyasagar M (1993). Nonlinear systems analysis.

[CR57] Wen J, Smith HL, Thieme HR (2016). Persistence versus extinction for a class of discrete-time structured population models. J Math Biol.

[CR58] Yakubovich VA, Leonov GA, Gelig AKh (2004). Stability of stationary sets in control systems with discontinuous nonlinearities.

